# Exosomes and Exosome-Inspired Vesicles for Targeted Drug Delivery

**DOI:** 10.3390/pharmaceutics10040218

**Published:** 2018-11-06

**Authors:** Sophia G. Antimisiaris, Spyridon Mourtas, Antonia Marazioti

**Affiliations:** 1 Laboratory of Pharmaceutical Technology, Department of Pharmacy, University of Patras, 26504 Rio-Patras, Greece; 2 Foundation for Research and Technology Hellas, Institute of Chemical Engineering, FORTH/ICE-HT, 26504 Rio-Patras, Greece

**Keywords:** liposomes, exosomes, extracellular vesicles, drug delivery, drug targeting, bioinspired systems, engineered systems

## Abstract

The similarities between exosomes and liposomes, together with the high organotropism of several types of exosomes, have recently prompted the development of engineered-exosomes or exosome-mimetics, which may be artificial (liposomal) or cell-derived vesicles, as advanced platforms for targeted drug delivery. Here, we provide the current state-of-the-art of using exosome or exosome-inspired systems for drug delivery. We review the various approaches investigated and the shortcomings of each approach. Finally the challenges which have been identified to date in this field are summarized.

## 1. Introduction

The most recent discoveries in the field of extracellular vesicles are unravelling exciting concepts on intercellular communication pathways [1,2]. The fast growing literature in this field is showing that nano to micron sized vesicles budding from cells and named exosomes (which are one of the types of extracellular vesicles) display specific organotropic behavior as part of their active role in cell-to-cell communication and material (protein and/or nucleic acid-cargo) transfer pathways. Besides local cell-to-cell communication, in some cases, the secreted factors play a key role in the interactions between cells located far apart from each other [3,4,5,6]. The tremendously high and specific organotropism of some types of exosomes is one of the major goals of all the types of nano-based drug delivery systems (DDSs) that have been screened to date, which remains unmet [7,8,9,10]. By providing important insights into the key elements that dictate the biological fate of vesicles and their ability to interact and be taken up by specific cells, the novel and fast-growing field of exosomes is now inspiring the design of ex-novo nanovesicles as targeted drug carriers for therapeutic applications. The exploitation of various types of exosomes or exosome (EX)-mimetics for targeted drug delivery is indeed a novel research area which is currently under intensive research [11,12,13,14,15,16,17].

In this review, we provide the current state-of-the-art on the development of exosomes, as well as artificial nanoparticulate systems that aim to mimic their properties (exosome-mimetics) as innovative nanocarriers with high drug targeting efficiency.

Due to the numerous similarities between liposomes and exosomes (which will be mentioned analytically in a separate part below), the application of liposome engineering technologies to engineer exosomes has been proposed as a way to overcome their limitations. The many similarities between the two systems is also the reason why liposomes are of the nanoparticle type, which is preferentially used for the construction of artificial exosomes or EX-mimetics as drug carriers. We will focus on such approaches since they may accelerate the development of specific roadmaps to construct engineered-exosomes or, the more realistic approach of developing exosome-mimetics, or else exosome-inspired liposomes as advanced targeted drug and/or imaging agent carriers in theragnostics.

### 1.1. Definition, Biogenesis and Main Functions of Extracellular Vesicles (EVs) and Exosomes (EXs)

In order to understand why exosomes can be used as targeted drug carriers, we need to initially clearly define what they are, and additionally, review their basic functions. Exosomes are one of the types of a broader category of cell-derived vesicles characterized as extracellular vesicles (EVs) [18,19].

**Scheme 1:**
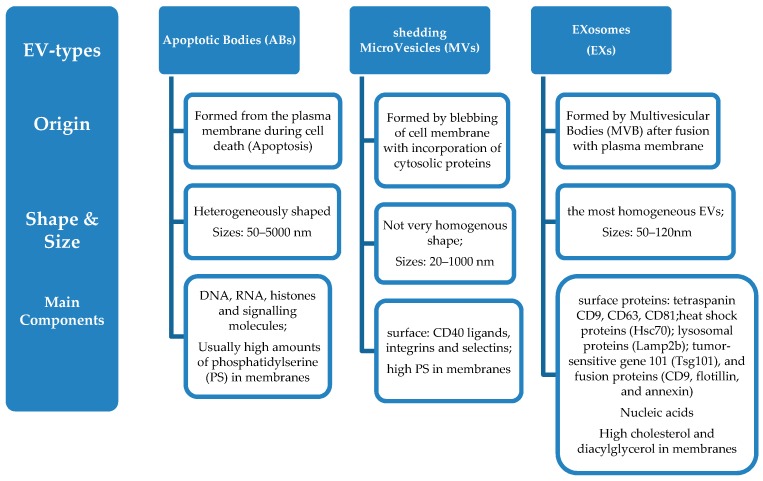
Types of extracellular vesicles (EVs) and characteristics. (i) Origin; (ii) Shape and Size distribution; (iii) Main components.

Extracellular vesicles are membrane-contained small vesicles, secreted by all types of pro- and eukaryotic cells [20,21]. The three main categories of EVs are (Scheme 1): (i) Apoptotic Bodies (ABs), (ii) Shedding Micro Vesicles (MVs) and (iii) Exosomes (EXs).

**Figure 1:**
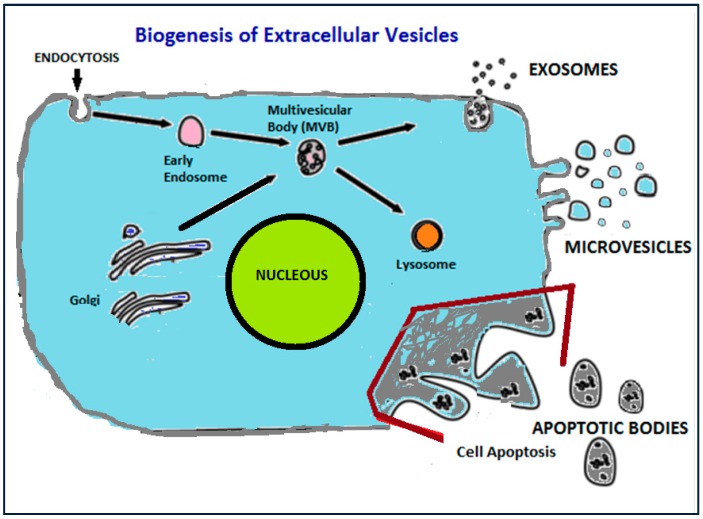
Representation of the biogenesis of extracellular vesicles from eukaryotic cell.

In more detail (Figure 1):

(i) Apoptotic bodies are released during cell death (apoptosis) and are heterogeneously shaped vesicles with sizes between 50–5000 nm. They are formed from the plasma membrane, and they contain DNA, RNA, histones, and signalling molecules [22]. They usually have high amounts of phosphatidylserine (PS) in their membranes, since the outer membrane of apoptotic cells is enriched in PS.
(ii) Micro vesicles are formed by blebbing of the cell membrane with concurrent incorporation of cytosolic proteins, and their sizes range between 20–1000 nm, depending on the origin cells and the method applied for their isolation from cell media [23]. Their formation can be triggered through Ca^2+^ influx, phorbol esters, ATP, etc. [24]. Some common biochemical characteristics have been identified between microvesicless from different cells, such as their high membrane levels of PS, and some common surface markers (CD40 ligands, integrins and selectins) [12].
(iii) Finally, exosomes include a more homogeneous (in shape and size) population of vesicles compared to microvesicles, with sizes that range from 50 nm up to 120 nm. Their biogenesis is initiated by inward budding of the plasma membrane which results in the formation of intermediate endosome-vesicles, the multivesicular bodies (MVBs). After that, depending on their composition, MVBs are either degraded after fusion with lysosomes or they fused with the plasma membrane and form exosomess that are released from the cells [25,26,27,28]. Exosomes contain surface proteins unique to the endosomal pathway, which are generally used to characterize exosomes and distinguish them from microvesicles, apoptotic bodies, and other vesicles such as tetraspanin CD9, CD63, CD81 [29], heat shock proteins (Hsc70), lysosomal proteins (Lamp2b), the tumor-sensitive gene 101 (Tsg101), and fusion proteins (CD9, flotillin, and annexin) [30], and incorporate nucleic acids, cytosolic proteins, and receptors. Their lipid composition differs from other extracellular vesicle-types, since they are rich in cholesterol and diacylglycerol. Exosomes are generally considered as transporters of miRNA that regulate specific intracellular mRNA activity [31].

As concluded from the definitions given above, microvesicles and exosomes are smaller compared to apoptotic bodies. Additionally they differ from apoptotic bodies in their content, since they rarely contain DNA [32]. The main function of microvesicles and exosomes is the intercellular transfer of lipids, RNA, and cytosolic proteins; however, it has been recently suggested that the two types of vesicles have different reporter molecule transfer functions [33,34].

Although these two vesicle-types, microvesicles, and exosomes, are separate classes of vesicles, due to the fact that they overlap in size, and since the commonly used non-specific protocols for exosome isolation and purification rely solely on the vesicle size differences (as described below), it is a fact that in many of the reports published, the exosome samples used are impure (since they probably also include microvesicles and large protein aggregates). Because of this, it has been proposed that the term “extracellular vesicles” (EVs) be used as a general term for all small vesicles/particles, including both vesicle types, and excluding only apoptotic bodies [22].

According to the later definition, one can state that extracellular vesicles are shed by most cells constitutively as well as in response to endogenous and exogenous triggers, and are involved in intercellular communication under both physiological and pathological conditions [32]. They mediate intercellular communication as signaling organelles (transmitting specific information from their origin-cells to their target-cells). In physiological conditions, extracellular vesicles are important mediators for cell-to-cell and inter-tissue communication, and exhibit important functions in regulating homeostasis as well as in a variety of other conditions [23,33,35]. In pathological conditions, the information transferred by extracellular vesicles, mainly cancer cell extracellular vesicles, may have detrimental effects. Extracellular vesicles have been demonstrated to contribute to various pathologies such as tumorigenesis and metastasis [36], inflammation [37], and immune system activation [38].

As a result of the above-mentioned functions, extracellular vesicles may serve as novel tools for various therapeutic and diagnostic applications, such as (a) anti-tumour therapy, (b) pathogen vaccination, (c) immune-modulatory and regenerative therapies and (d) drug delivery.

Herein, we will focus on the application of extracellular vesicles in drug delivery, and present analytically the attempts that have been made to use exosomes (or extracellular vesicles) for drug delivery [14]. Indeed, in the last 5 years, extracellular vesicles were proven to have high targeting potential for specific cell types, but in most cases, following systemic administration, they failed to demonstrate the anticipated therapeutic results [14]. The later failed attempts revealed a number of shortcomings in the methodologies employed for using extracellular vesicles as targeted-DDSs. It is well-known today that the main pre-requisites for using any type of vesicle for the targeted delivery of drugs are that: (i) they can be loaded with a sufficient amount of drug, in order to elucidate a therapeutic response; (ii) they are stable (retain their size, structure, and drug load) during circulation in the blood stream before reaching their therapeutic target in the body; (iii) they can avoid uptake by the macrophages and circulate for prolonged time periods so that they can reach their cellular targets; and (iv) they are biocompatible, or else non-toxic and non-immunogenic [39]. In accordance to these requirements, the main problems identified (and still remaining to be solved) for the successful translation of extracellular vesicles into targeted drug carriers are the following: (i) drug loading and/or retention of drugs in exosomes is not sufficient; (ii) the poor pharmacokinetics of natural extracellular vesicles when loaded with bioactive agents, and (iii) the fact that a big problem for such systems to be included in industrial roadmaps still remains, giving very low yields of isolation from biological media or cell cultures (see paragraph 3, below).

Due to the aforementioned shortcomings, other approaches are currently being exploited, such as the use of exosomes- or extracellular vesicle-mimetics, which are, in most cases, liposomes constructed after appropriate selection of their components in order to mimic the composition (and hopefully) the structure of organotropic extracellular vesicles.

Before ending this introductory part, we should mention that some attempts have been made to date in order to classify the various types of extracellular vesicles, and some general databases such as Vesiclepedia (http://microvesicles.org/) and Exocarta (http://www.exocarta.org/) have been developed. Nevertheless, as mentioned, due to the technical difficulties encountered when attempting to purify and accurately identify each vesicle-type, it is not clear in many of the past studies which specific kind of vesicles are actually used; thus, the use of the terms microvesicles and exosomes is not consistent in many of the previously published reports.

### 1.2. Current Bottlenecks in Nanoparticle-Assisted Targeted Drug Delivery and Liposomes

Several types of nano-based drug delivery systems (DDSs) have been, over the last 4 decades, and are currently being considered for drug targeting applications. Among all the nano-based DDSs, liposomes—that first reached clinical approval—are the most biocompatible and least toxic artificial systems, which is logical since they are constructed by phospholipids and cholesterol, the main components of cell membranes [39].

Liposomes have made great impact on therapeutics due to their advantages as DDSs. Indeed, in addition to their non-toxic and biocompatible nature that can accommodate high payloads of drugs, they have the capability of loading multiple drugs in order to provide protection of drugs from degradation, and to enhance drug endocytosis into cells. It is well known that Liposome-assisted drug delivery has a major impact on many biomedical areas, and that several liposomal formulations are proven to stabilize therapeutic compounds, overcome cellular/tissue uptake obstacles, and improve biodistribution, enabling thus the effective delivery of encapsulated drugs to target sites and minimizing their systemic toxicity [40]. Liposomes are particularly potent in the treatment of some types of cancer, in which they can achieve passive targeting via the leaky tumor vasculature, according to the enhanced permeability and retention (EPR) effect. As a result of all the advantages of liposomes as DDSs, several liposomal drug products are currently available in the market, such as Doxil®, Ambisome®, DaunoXome®, Marqibo®, Onivyde™, Inflexal® V, etc., while others are under clinical testing, such as an amikacin liposomal formulation, the Stimuvax® liposomal vaccine, Liprostin™ a prostaglandin E-1 liposome formulation, etc. However, despite efforts to increase the active targeting of drugs to diseased sites by incorporation into ligand-targeted-liposome formulations [41,42,43], none has been successfully translated into products [39], leading to a bottleneck that has limited their broader applicability in therapeutics.

In the last 30 years, the use of numerous targeting ligands of different types (monoclonal antibodies, proteins, peptides, small molecules, carbohydrates, aptamers etc.), to target different receptors which are overexposed on the membrane of the target cells (such as HER2, VEGF, e-selectine, nucleolin, tranferrin, folate and EGR receptors, V3 integrin, SSTR2, ASGPR, etc.), has been investigated with the aim of further increasing the delivered fraction of liposome-encapsulated cargo to diseased sites, compared to the amount delivered via (control) non-ligand-targeted liposome formulations [44]. However, despite improved biodistribution and therapeutic outcomes in preclinical studies, the translation of such ligand-targeted liposome formulations into the clinic has not been successful. Several possible reasons, which perhaps have not been adequately considered, have been proposed to explain the former discrepancy, such as (i) disease-dependent anatomical barriers, (ii) target accessibility and expression, (iii) formulation stability, and (iv) density of the targeting-ligand(s) on the liposome surface [44].

In addition to the bottlenecks of insufficient targeting efficiency and translation difficulty due to their complexity, which have been identified for ligand-targeted liposomes, other types of targeted nanoparticles also suffer from poor biocompatibility and biodegradability, as well as immunogenicity (which may also be a problem for some types of targeted liposomes), factors that could be resolved by exosome-based drug delivery systems. Currently, the need for superior targeted-drug carriers is further increased due to the fact that the tremendous therapeutic potential of biopharmaceuticals will become available only after formulation and delivery issues are resolved. Biopharmaceuticals (therapeutic peptides, proteins and nucleic acids) demonstrate exquisite specificity which enables successful treatment of serious unmet medical needs; therefore, they represent a growing fraction of the current drug development pipelines [45].

Additionally, the development of drugs that act on the central nervous system is presently severely hampered by the lack of efficient methods to deliver the drugs (especially biophramaceuticals) into the brain. It is well known today that only a very small fraction (<1%) of injected antibodies enter the brain by passive diffusion, while other large molecule-drugs (e.g., peptides) can be only administered by peripheral injection or invasive intra-cranial procedures, approaches that failed in the clinic. The extremely challenging issue of blood-brain-barrier (BBB) crossing is an urgent need, taking into consideration the worldwide rise in neurodegenerative disorders, as well as the yet unsolved problem of treating brain cancers, both situations having at present no recognized therapeutic solution [46].

In addition to the approaches mentioned above, other advanced methodologies have been exploited with the aim of further increasing the targeting potential of ligand-targeted-liposomes [9,47]. In this context, phage display methodologies have been utilized for the selection of high affinity ligands [48]. Additionally, multi-targeted liposomes which can target two of more receptors simultaneously have been constructed, with the aim of increasing liposome targeting efficiency [49]. Other approaches employ the use of physical stimuli to further increase the targeting efficiency of ligand-targeted-liposomes, such as magnetic- or ultrasound-enhanced targeting [50,51,52]. Nevertheless, in addition to other potential problems, the multifunctional systems may be too complicated for translation into drug products, a factor that should also be seriously taken into account when seeking solutions to the bottelnecks of actively-targeted liposomes, or nanoparticles in general).

Along this line, the recent findings demonstrating the high potential of engineered-extracellular vesicles for successful delivery of bioactive agents to selected body targets open new horizons towards the development of liposomes with compositions inspired from extracellular vesicles (extracellular vesicle-inspired liposomes) that will hopefully demonstrate enhanced targeting efficiencies, compared to the ligand-targeted-liposome types which have been exploited to date.

## 2. Similarities and Differences between Exosomes and Liposomes

**Figure 2:**
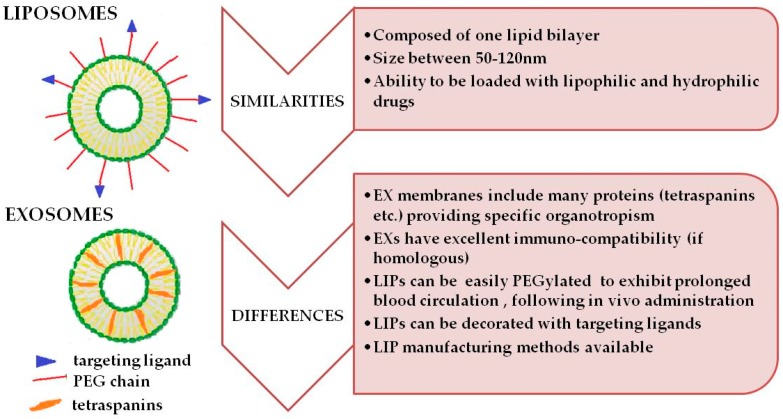
The basic structural characteristics, as well as the main similarities and differences of liposomes and exosomes are presented.

As reported before [14,53], exosomes have many similarities with small unilamellar, vesicle-(SUV)-type liposomes (Figure 2), which explains why most types of artificial exosome-mimetics are mainly based on liposomes. These similarities could allow researchers to engineer exosomes (in order to load drugs into them, and/or attach different molecules on their surface), using the methodologies developed in the liposome technology field. Indeed, both exosomes and SUV-liposomes, are vesicular structures consisting of one lipid bilayer, with mean diameters ranging from 50 nm to 120 nm. The major difference between SUV-liposomes and exosomes is the complex surface composition of exosomes, and more specifically, the characteristic array of membrane proteins such as tetraspanins, which are present on the membrane of exosomes, whereas SUV-liposomes do not usually have proteins in or on their lipid bilayer. These exosomal proteins are required for facilitation of their efficient targeting and uptake by recipient cells. However, one should not only focus on the protein components of the exosome surface, ignoring their unique lipid composition, since this may play an important role in the incorporation and functional conformation of the proteins in their membrane, as well as in the specific interactions that will occur between exosomes and serum proteins, after their administration, which will dictate the in vivo fate of the vesicles.

**Scheme 2:**
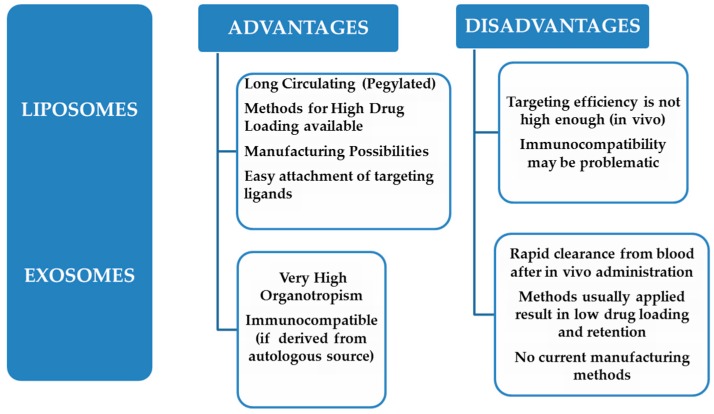
Advantages and disadvantages of liposomes and exosomes.

When comparing the advantages and disadvantages of exosomes and liposomes, with respect to their applicability as targeted-DDSs, it becomes evident that the two systems are complimentary, since the advantages of the one system are disadvantages of the other and vice-versa (Scheme 2). Thereby, the incorporation of the advantageous features of the two vesicle types into one hybrid vesicle, if possible, would most probably result in the realization of an advanced system for drug targeting applications. Such approaches have indeed been initiated [54], and will be described in detail below.

Due to their similarities with exosomes, SUV-liposomes are usually engaged as control vesicle formulation for comparison to detect the superiority of exosomes for drug delivery applications. However, some previously reported head-to-head comparisons of exosomes versus liposomes have recently been weakened due to the inadequate choice of the liposomes that were used as controls. Researchers are thus encouraged to implement more appropriate controls in order to prove any potential superiority of exosomes over liposomes or other synthetic nanoparticles [55].

## 3. Sources, Methods of Isolation and In Vivo Clearance of Unmodified Exosomes

In this part, brief reference to the sources of extracellular vesicles and exosomes will be made, since the parental cells from which the vesicles are extracted strongly determines their structural components, and thereby, plays the most important role on their functions and potential in vivo fate. Additionally, the methods used currently for isolation and purification of exosomes will be described, as well as the basic findings from studies that have been conducted to date for exploitation of their in vivo kinetics and their clearance from the blood circulation following their in vivo administration via various administration routes.

### 3.1. Sources of Exosomes

Most cell types such as dendritic cells (DCs), epithelial cells, macrophages, reticulocytes, mast cells, platelets, neurons, B cells, T cells, oligodendrocytes, tumour cells, and Schwann cells have been demonstrated to be able to release exosomes [29,31,32,33,56,57,58]. Most of the in vivo-detected circulating exosomes (about 80%) are derived from platelets [32,58].

Mesenchymal stem cell (MSC)-derived exosomes are currently being exploited for numerous applications since they have been shown to play a role in cell-free therapy of many diseases, including myocardial infarction, drug addiction, and status epilepticus [59]. They are also thought to be able to ameliorate liver injury, inflammation-induced preterm brain injury, and various types of cancer.

In addition to the aforementioned cells, exosomes are also found in many physiological fluids such as plasma, saliva, urine, lymph, breast milk, semen, amniotic fluid, ascites fluid, bronochoalveolar lavage fluid, cerebrospinal fluid, bile, malignant pleural effusion, etc. [33,60,61,62,63,64,65,66,67].

The cellular origin of exosomes strongly determines their contents, and as a consequence, also their functions. Exosomes which are produced by B lymphocytes contain functional MHCI, MHCII, and T cell co-stimulatory molecules, and can thus stimulate T cell proliferation [68]. Alternatively, exosomes from cancer cells contain cell adhesion related molecules, such as gelatinolytic enzymes, and thus, they have the ability to enhance the progression and metastasis of tumors [69]. Cancer cell-derived exosomes are actively incorporated by mesenchymal stem cells (MSCs) (in vitro and in vivo), since the transfer of exosomal proteins and miRNAs depends on the physical and functional characteristics of tumor-supporting fibroblasts [70,71].

For further information about signal transmission pathways and molecular contents of exosomes, which are beyond the scope of this review article, readers may refer to ExoCarta (http://www.exocarta.org) or EVpedia (http://evpedia.info), as well as the American Society for Exosomes and Microvesicles (http://www.asemv.org).

In reference to current efforts to use exosomes as drug delivery systems, it should be highlighted that despite the identified very high organotropism of cancer cell-derived exosomes, a shift to safer alternatives such as exosomes produced from foods has been recently made in this research field. From all the relevant sources of this category, bovine milk- and fruit-derived exosomes are probably the most investigated for drug delivery [72]. Indeed, bovine milk has been proposed as a scalable source of exosomes that can act as a carrier for chemotherapeutic/chemopreventive agents [73]. In addition to bovine milk, exosomes from milk from other sources, such as porcine and murine milk, have also been considered [74].

### 3.2. Isolation Methods

Several methods have been developed to efficiently isolate exosomes from cells and biological fluids [75]. Each method exploits a specific trait of exosomes, such as their size, shape, density, or surface antigens to aid isolation. However, the most challenging task of all methods employed is to specifically purify exosomes from the wide spectrum of extracellular vesicles, cellular debris, and interfering molecular components [76,77]. For this reason, the quality of each batch of isolated exosomes should be examined before any further application is persued. Several techniques have been used to measure exosome size, size distribution, morphology, quantity as well as protein and RNA composition [78].

In all the exosome isolation methods described in the literature, the first general step is to culture parental cells in serum-free media and allow the cells to condition the media. Conditioned media is then collected and processed in different ways based on the specific isolation method applied [76,77]. For bodily fluids or other types of fluids, the principle for exosome isolation is the same as when starting from cell culture conditioned medium, but because of the vicosity of some fluids, it is necessary to dilute them, and in some cases, to pre-clean them from large bioparticles, as well as to spike them with protease inhibitors in order to prevent potential degradation of the exosome proteins [57].

#### 3.2.1. Ultracentrifugation

Ultracentrifugation (U/C)-based exosome isolation is considered as the gold standard, and is one of the most commonly used and reported techniques for exosome isolation [75,76,77]. There are two types of methodologies based on the principles of separation: differential and density-gradient U/C. Differential U/C involves serial stepwise centrifugations in order to isolate the exosomes from other components in the sample based on their density and size differences. Density-gradient U/C segregates exosomes according to their size, mass, and density to specific layers in a gradient material such as sucrose. Usually, at the final step, small exosomes are isolated and resuspended in phosphate buffer saline (PBS), as this is the most commonly-used solvent in all applications [75,76,77]. As U/C is mostly based on vesicle size, the purification of exosomes from other extracellular vesicle-types, and mainly from microvesicles that have overlapping size distributions with exosomes, is questionable.

#### 3.2.2. Immunoaffinity

Immunoaffinity capture-based techniques exploit interactions between antibodies and selective exosome surface proteins in order to isolate them [76,77]. Higginbotham et al. recently demonstrated the feasibility of using fluorescence-activated vesicle sorting to analyze and sort individual exosomes from DiFi cells (human colorectal cancer cells). Indeed, the endothelial growth factor receptor (EGFR) and the exosomal marker CD9 were detected on the surface of DiFi exosomes by mAb conjugation [79]. Ideally, the exosomal tags for immunoisolation should be membrane-bound, lacking soluble counterparts, and should of course be highly expressed on the surface of the exosomes produced by the specific biological source selected. According to the applied immunoaffinity procedure, antibodies which are specific for the surface proteins of the exosomes to be isolated (e.g., CD63, CD81, CD93 etc.) could be immobilized on a variety of support media, such as magnetic beads, chromatography matrices, and elisa plates, as well as microfluidic devices [75,80].

Even though this finally results in a comparable production yield when compared to U/C methods, immunoaffinity uses much less sample-volume and extracts larger amounts of purified exosomes, regardless of the vesicle size differences.

#### 3.2.3. Other Size-Based Isolation Methods

The most popular size-based exosome isolation technique is ultrafiltration. Based on their size, exosomes can be isolated with sequential filtration using membrane filters with defined size-exclusion limits (specific membrane pore sizes) [81].

Another size-based separation technique is size-exclusion chromatography that uses a column packed with beads that have smaller pores to those of the exosomes. Fractions are eluted sequentially in order of decreasing sizes, and exosomes can be thus isolated, from larger and smaller particles.

Size-exclusion procedures are less time-consuming compared to U/C methods, and furthermore, do not require special equipment (ultracentrifuge). However, biologic fluids and cell culture media contain a large number of nanoparticles in the same size range to that of exosomes [77]; thereby, such size-exclusion methodologies could only be applied when the aim is to obtain mixed extracellular vesicle samples, rather than pure exosomes.

#### 3.2.4. Precipitation

Exosomes can be isolated from biological fluids by altering their solubility. Various methods use polymers that can precipitate exosomes according to their surface characteristics. Specifically, by binding with water molecules, the polymers force the less soluble exosomes out of the solution, and the exosomes can be then collected by low speed centrifugation [76,77]. In several biologic fluids, precipitation seems to realize higher exosome recovery compared to U/C. However, a big disadvantage of this method is the co-precipitation of other non-exosomal contaminates, including the polymeric material used [76,82].

#### 3.2.5. Yield of Common Isolation Methodologies

Despite the impressive progress that has been made in exosome isolation in the recent years, it has been proven to be quite challenging to rapidly and efficiently isolate exosomes, due to the heterogeneity of extracellular vesicles, the complexity of the biological fluids used, and the potential contamination of the starting material from other extracellular components with similar sizes and biochemical characteristics. Many isolation methods are currently available, as analyzed above. A commnon aspect among these methods is that they all involve multistep procedures, and thus, they finally provide a low production yield and/or a low purity of exosomes. The U/C method for instance, which is currently the most classical and reliable isolation technology, can isolate only a small portion of extracellular vesicles (~20–25%). On the other hand, immunoaffinity methods require expensive antibodies and matrices, but finally lead to similar yields to those acquired when using U/C. Size-exclusion methods are often used in combination with U/C or other techniques, but the complex-methods finally realize low yields due to the fact that a large fraction of the (extracellular vesicle) sample is lost due to its adhesion to the gel materials or the filters. Polymeric precipitation instead might achieve a higher yield than the other methods, but cannot purify the exosomes from the polymeric material used [83,84]. Altogether, the very low production yield of exosomes imposes a tremendous impediment to their utility in research, thus delaying their potential clinical translation for any type of therapeutic application.

Several strategies have been empirically employed and reported in the literature to circumvent poor exosome production yield. These include prolonging incubation times to increase the secretion of exosomes from the cells, applying higher initial sample density, or changing the composition of the culture medium. In a recent report, when heat stress was applied to MCF-7 cells, it was shown to increase the number of exosomes produced by the cells (compared to the same number of non-stressed cells) [85]. However, none of these approaches have resulted in any significant increase of exosome production yield or purity. Thus, a novel approach to substantially augment the production yield of exosomes is urgently needed.

#### 3.2.6. Microfluidic Methods for EX/EV Purification

The use of microfluidics has been recently explored in the search for improved technologies for exosome isolation and purification. Wu et al. developed a unique lab-on-chip technology that integrates acoustics and microfluidics to isolate extracellular vesicles directly from biological fluids, such as whole blood, with high purity and yield. This acoustofluidic device consists of two modules that are intergrated into a chip: a microscale cell-removal module that first removes larger components, followed by an exosome-isolation module that separates the different types of extracellular vesicles. It is an automated exosome isolation method with short processing time that offers the advantage of the preservation of the structure, characteristics, and functions of exosomes [86]. Another label-free passive microfluidic approach recently implemented for isolating exosomes is viscoelastic microfluidics, where particle separation is determined by elastic lift forces acting on particles of different sizes in a viscoelastic medium [87]. The microfluidic chip uses a viscoelastic sheath fluid to align the EV sample along the sidewalls of the channel; then, exosomes can be isolated from two-sided outlets. This device achieves >90% purity and >80% recovery of extracellular vesicles from cell culture media.

Another interesting method utilizes a three-dimensional nanostructured microfluidic chip to isolate extracellular vesicles in anefficient way. This device consists of ciliated micropillars, forming a porous silicon nanowire-on-micropillar structure. It was demonstrated that this microfluidic prototype device can preferentially trap extracellular vesicles, while simultaneously filtering out proteins and cell debris. Trapped vesicles can be recovered intact by dissolving the porous nanowires in PBS buffer [88]. In another study, an alternative microfluidic-based device has been developed for the isolation of extracellular vesicles from biological fluids. Specifically, the developed device was an integrated double-filtration microfluidic platform that was shown to efficienlty isolate, enrich, and characterize extracellular vesicles from urine samples of healthy volunteers and cancer patients. This method opens up new avenues in clinical diagnosis and therapeutic outcome monitoring, since the vesicles isolated from the biological fluids could be used for cancer diagnosis and for monitoring the efficiency of different treatments [89].

Recent technological progress in exosome isolation with microfluidic-based techniques has increased the efficiency of exosome isolation. However, the microfluidic devices described above need further validation and standardization before they can be translated into devices for clinical diagnosis and treatment.

### 3.3. In Vivo Clearance of Unmodified Exosomes

As briefly mentioned above, one of the initial shortcomings identified for using exosomes as carriers for drug targeting applications is their rapid clearance form circulation and very short residence time in the blood following in vivo administration. Indeed several types of extracellular vesicles from different sources have been administered in vivo in order to follow their biological fate.

In one study, unmodified tumor-derived exosomes isolated from the supernatant of 4T1, MCF-7, and PC3 cells together with PC/Chol liposomes (studied as control vesicles) and also liposomes formulated with the lipid extracts of the exosomes (exosome-mimetics), were found to demonstrate a comparable rapid clearance and minimal tumor accumulation following i.v. injection. These findings indicate that the unique protein and lipid composition of the exosomes do not appreciably alter their clearance rate or their biodistribution, following i.v. administration [90]. However, it was interesting that when delivered intratumorally, the exosomes remained associated with tumor tissue to a significantly higher extent compared to PC/Chol liposomes [90]. In another pharmacokinetic study, it was found that most of the administered exosomal dose did not reach the target tissue but was sequestered in other tissues, while the spleen was found to have a major role in exosome clearance [91]. Sun et al. found that 1 h post- intraperitoneal injection of curcumin-loaded exosomes in mice, the vesicles accumulated in the liver, lungs, and spleen [92]. A similar high accumulation in the liver and spleen was also observed by Hwang et al. [93] with 99 m Tc- hexamethylpropyleneamine oxime (HMPAO)-labeled exosomes. Takahashi et al. observed that luciferase-expressing exosomes (produced from transfected B16 cells) rapidly disappeared from the blood (half-life of 2 min) after i.v. injection in mice, because they were sequestered in the lungs and the spleen [94]. Morishita et al. measured the tissue biodistribution of 125 I-labeled exosomes isolated from transfected B16-BL6 cells following their i.v. injection in mice, and found that 4 h post-injection, 28%, 1.6% and 7% of the radioactivity was detected in the liver, spleen, and lung, respectively, while it was additionally proven by other studies that exosomes are mainly captured by macrophages [95,96].

Phagocytosis has been identified as the principal mechanism of exosome internalization, and different mechanisms for exosome endocytosis in macrophages have been described [97,98]. Integrins and lactadherin that binds to αvβ3/β5 integrins which are expressed in exosomes from dendritic cells, seem to have an important role in exosome capture [99]. Additionally, the deposition of fragments of the complement protein C3 on the surface of exosomes as well as the lectin galectin-5 found on red cell-derived exosomes, seem to be involved in macrophagic capture. Convincing evidence was also reported about the capture of exosomes by a specific subpopulation of macrophages, the CD169+ macrophages, via their interaction with the sialylated protein receptor CD169 [91]. The spleen has also been identified as a key anatomical station for exosome sequestration, and a clarification of the mechanisms involved in this process will be required in order to develop modified versions of exosomes that will be able to escape splenic capture and demonstrate increased biological activity [91].

In contrast, some types of allogenic exosomes have been demonstrated to realize a privileged immune status, and demonstrate decreased clearance by MPS. Specifically, exosomes derived from immunocytes express the CD47 receptor [100] that interacts with signal regulatory protein α (SIRPα) resulting in the generation of a signal which is characterized as the “don’t eat me” signal, that blocks their uptake by phagocytes [101]. Exosomes released by primary fibroblast-like mesenchymal cells can also bypass immune clearance by monocytes and macrophages utilizing a similar mechanism, and demonstrate prolonged blood circulation [102]. Thereby, the source of exosomes seems to be particularly important conserning their clearance kinetics and mechanisms, and as a consequence, probably also their biodistribution.

In any case the problem of rapid clearance could be solved by the introduction of polyethylene glycol (PEG) molecules to coat the surface of the vesicles, as successfully applied for many years in the liposome field. In fact, Kooijmans et al. have recently shown that the introduction of PEG to exosomes, with a method which is similar to that used for liposomes (post-formation incubation with PEG-lipid micelles), results in stealth properties, which significantly increases the circulation time of exosomes in mice [103].

In conclusion, the currently-available knowledge concerning the in vivo disposition and blood circulation of unmodified exosomes reveals that in most cases (depending of the exosome source), the need for ways to engineer their surface characteristics in order to increase their circulation time may be required (depending of course on the specific application and administration route). Such approaches may increase the potential applications of exosomes for drug targeting applications, and will be discussed in the next part.

## 4. Types of Systems

**Scheme 3:**
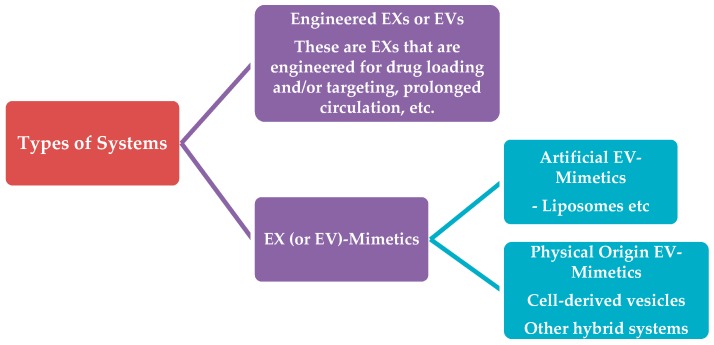
Classification of EV-like vesicles used for drug delivery applications.

In this review, we will use the following categorization and naming for the various types of exosome-like systems which have been tested to date as drug carriers (Scheme 3). The two main categories of exosome-evolving-vesicles that are being currently used for drug delivery will be defined as:

(1). Engineered-Exosomess or Extracellular Vesicles. This first category will include vesicles derived from isolated and purified exosomes or extracellulat vesicles. In order to be used for drug delivery applications, exosomes are initially engineered for:

- The efficient loading of drugs and/or
- Surface-modification/attachment of molecules on their surfaces; such modifications may be required when the in vivo fate (stability and/or pharmacokinetic/biodistribution profile) of the exosomes is not considered to be adequate for the planned drug delivery and/or targeting application. In fact, as analyzed above, in most cases, the clearance of unmodified exosomes after their in vivo administration (especially if iv-injection is used) is rapid, posing a problem for their applicability as targeted drug carriers.

(2). Exosome or Extracellular Vesicle-mimetics. This second category of vesicles includes all the vesicle-types that are not formed using exosomes or extracellular vesicles as their starting material. Such approaches have been explored in order to overcome the low yields of exosome isolation/purification methodologies, or to develop drug carriers for broader applicability (since the use of non-autologous cellular materials or material of tumour origin could trigger numerous types of toxicities due to immunological responses or other reasons). Thereby, exosome-mimetics are further divided in sub categories, based primarily on the origin of the starting materials used for their preparation. Here, we distinguish two sub-categories of exosome-mimetics:

- Artificial Exosome-mimetics, when the starting material is of synthetic or semi-synthetic origin (this category also includes lipids extracted from natural sources, such as cells or extracellular vesicless). In most cases, artificial exosome-mimetics are actually liposomes with or without specific proteins in their membrane (which are inspired from specific types of exosomes with high organotropism).
- Physical-origin exosome-mimetics, when the starting material may be derived from other types of cellular components excluding extracellular vesicles, such as whole cells (in this case the vesicles are names “cellular vesicles”). In this subcategory, again, the starting material is engineered as mentioned above for engineered-exosomes, and the same methodologies apply.

In this part, we will present examples of the various types of systems, categorized as mentioned above, and we will additionally describe the methods used up to now to load drugs and modulate the surface of the various types of vesicles.

### 4.1. Engineered-Exosomes or Extracellular Vesicles

Several studies have exploited the potential use of extracellular vesicles for drug delivery/targeting, or, in general, for theranostic applications, after being engineered for drug loading, surface modification, or both. Some recent examples are mentioned here.

Pascucci et al. prepared paclitaxel (PTX)-encapsulating exosomes that demonstrated strong anti-proliferative activity in vitro against CFPAC-1 human pancreatic cells [104]. The feasibility of PTX-exosomes as a potent chemotherapeutic system to treat MDR cancer was also assessed, and PTX-exosomes demonstrated 50 times higher cytotoxicity towards drug resistant MDCKMDR1 (Pgp+) cells (compared to the control formulations). In another study, a potent anticancer effect by exosomes was observed in a model of murine Lewis lung carcinoma pulmonary metastases [105]. Lv et al. reported that extracellular vesicles isolated from resistant anticancer drug-treated HepG2 cells conferred superior immunogenicity in inducing HSP-specific NK cell responses, a finding that could lead to the development of an efficient vaccine for hepatocellular carcinoma immunotherapy [106]. Sun et al. investigated the potential to deliver curcumin, a polyphenol with anti-inflammatory and antineoplastic activity, by mouse lymphoma-derived (EL-4) exosomes [92]. In another study, curcumin-loaded EL-4-derived exosomes were efficiently noninvasively delivered to microglia cells via the intranasal route [107]. Haney et al. successfully used RAW264.7 cell-exosomes to deliver catalase (a tetramer protein of 250 kDa), and the engineered-exosomes were readily taken up by neuronal cells in vitro, while a considerable amount of the same catalase-loaded-vesicles were detected in the brain of a Parkinson’s disease mouse model after intranasal administration, and realized a significant neuroprotective effect [108]. In another study, exosomes were engineered for both Doxorubicin (DOX) loading and RGD peptide attachment on their surface. These dually-engineered exosomes presented highly efficient targeting of DOX to αv integrin-positive breast cancer cells in vitro, while they were found efficient for targeted delivery of DOX to tumors after i.v. injection, leading to a significant inhibition of tumor growth without demonstrating any measurable toxicity [109]. The later example proves the potential of specific exosome-types to target tumors even after i.v. injection, although as presented above, unmodified exosomes are usually rapidly cleared from blood circulation.

Nakase and Futaki encapsulated a dextran macromolecule (70 kDa) into CD63-GFP-HeLa cell-derived exosomes. The exosomes were engineered to combine a cationic lipid (Lipofectamine) and a pH-sensitive fusogenic peptide (GALA) that would induce the fusion of endosomal and exosomal membranes for improved uptake by cells. Indeed, the cellular uptake of dextran by the engineered-exosomes was significantly enhanced by the combined treatment [110]. Fuhrmann et al. engineered exosomes so as to encapsulate different types of porphyrins (with differing hydrophobicities), and such exosomes were demonstrated to induce a stronger phototoxic effect in a cancer model compared to the free drugs [111].

The delivery of small RNAs using engineered-exosomes has been studied by several groups [112,113,114,115,116]. In more detail:

Alvarez-Erviti et al. engineered dendritic cell-exosomes to be loaded with exogenous siRNA and have RVG peptides attached to their surface. After iv-injection, these RVG-targeted exosomse were observed to delivere siRNA specifically to neurons, microglia, and oligodendrocytes in the brain [112], proving again the targeting ability of surface modulated-exosomes after i.v. administration.

Peripheral blood plasma derived exosomess were used to transport exogenous siRNA to human blood cells, and siRNA was successfully delivered into monocytes and lymphocytes, causing selective gene silencing of mitogen-activated protein kinase 1 [113].

In another study, extracellular vesciles loaded with siRNA were sucesfully delivered to recipient cells, reducing HER2 protein expression [114].

Didiot et al. demonstrated that exosomes that were loaded with hsiRNAs targeting *Huntingtin* mRNA were efficiently internalized by mouse primary cortical neurons, and promoted dose-dependent silencing of *Huntingtin* mRNA and protein [116].

Nevertheless engineered-exosomes have also been found to perform worst compared to some liposome-types. Indeed, exogenous cholesterol-conjugated siRNAs (Chol-siRNA) and endogenous miRNA were inserted in exosomes from both, a melanoma and a monocyte/dendritic cell (DC) line, and their delivery potential in distinct target cells was assessed. The delivery of siRNA by the engineered-exosomes and also by anionic fusogenic liposomes (prepared by employing the same loading approach, as control formulations), was tested; the results showed that the exosomes were unable to functionally deliver the associated small RNAs. In contrast, the anionic fusogenic liposomes induced a marked siRNA-mediated gene knockdown under identical experimental conditions [117].

Recently, macrophage-derived exosomes were engineered to attach on their surface a PEG-conjugated ligand targeting the Sigma receptor, and they were additionally loaded with PTX; they were found to exhibit superior in vitro and in vivo results compared to the control formulations against a pulmonary metastases model [118].

### 4.2. Exosome (or Extracellular Vesicle)-Mimetics

As mentioned above, there are two types of Extracellular Vesicle-mimetic systems: (a) Artificial exosome-mimetics and (b) Physical-origin Extracellular Vesicle-mimetics. The main theoretical basis, and some examples of the potential applications for drug delivery of the two different types, are presented below.

#### 4.2.1. Artificial Extracellular Vesicle-Mimetics

While pure populations of exosomes can be isolated from exosome-secreting cell lines, these exosomes, unlike those released from autologous primary cells, have immunogenic and oncogenic potential, inhibiting their broad use as drug delivery systems. Moreover, extracellular vesicless play multifaceted roles in health and disease, including the intercellular transfer of pathogens and disease-associated proteins [119,120], introducing major barriers for the translation of naturally secreted exosomes to the clinic. Extracellular vesicle-mimetics may help circumvent these barriers [53,121].

Artificial extracellular vesicle-mimetics are based on the idea that not all components in natural exosomes are essential for specific and efficient delivery. Thus, assembling lipids into a bilayer structure (which resembles the membrane of the exosome) and functionalizing the vesicle surface with proteins, or modulating their surface by the transport of a message through direct contact with target cell receptors, or by attaching hydrophilic molecules to increase their blood circulation, is considered as an artificial extracellular vesicle-mimetic. As mentioned above, most of the artificial extracellular vesicle-mimetics proposed or studied to date are actually liposomes. Theoretically, by using the knowledge acquired by appropriate analysis of the surface characteristics of organotropic extracellular vesicle-types about their composition, one may be able to develop artificial liposomal systems with the desired targeting properties. Proteomic and lipidomic analysis may be helpful to identify the most important extracellular vesicle components that determine their high targeting potential, and elucidate their structure in order to make it possible to develop liposomes as artificial extracellular vesicle-mimetics. Importantly, only small unilamellar vesicles (SUVs) are ideal precursors for the preparation of vesicles that can mimic exosomes due to their similarities to natural exosomes (size range and membrane disposition). Thus, by applying classical techniques used for preparation of SUV liposomes (e.g., thin-film hydration method, reverse-phase evaporation method, ethanol injection method, ether injection method, microfluidic-based methods, extrusion techniques, etc.), liposomes with a size range similar to that of natural exosomes can be easily obtained.

Some examples of such artificial exosome-mimetics developed for drug delivery applications follow:

Very recently, exosome-mimicking liposomes (formulated by copying the lipid composition of exosomes as a starting point) were tested for the delivery of VEGF siRNA to A549 cancer cells and HUVECs. These exosome-mimetics had lower cytotoxicity compared to Lipo-2000 and DOTAP liposomes, and higher storage and physical stabilities (reduced aggregation) in the serum. They also appeared to be able to be endocytosed into A549 cells and HUVECs. Notably, these exosome-mimicking liposomes exhibited significantly higher cellular uptake and silencing efficiency compared to PC/Chol liposomes. However, their oligonucleotide delivery efficiency was still very low compared to that of cationic lipids, such as Lipo 2000 and DOTAP [122].

The following examples are not directly related with artificial-exosomes as drug delivery systems but as therapeutics; however, they are of interest, since the results prove that the artificial exosomes can target specific cell types.

In one study, targeted and in vivo traceable artificial exosomes were developed to mimic dendritic-cell-derived exosomes. The theoretical background is that dendritic-cell-derived exosomes are known to mediate and modulate immune responses in vivo by semi-direct T cell activation, and that T cells can eradicate primary, metastatic, and relapsed tumors and ameliorate otherwise fatal viral infections. Taking this into account, the exosome-mimetic-liposomes were coated with an optimized number of MHC Class I/peptide complexes and a selected specific range of ligands for adhesion, early activation, late activation, and survival T cell receptors. It was finally shown that that these artificial-exosomes activated and expanded the functional antigen specific T cells at sufficient levels [123].

The group of Martinez-Lostao et al. observed that T lymphocytes, which were present in synovial fluid (SF) of rheumatoid arthritis (RA) patients, were sensitive to APO2L/TRAIL. In addition, there was a drastic decrease in the amount of bioactive APO2L/TRAIL associated with exosomes in SF from RA patients [124]. Taking these findings into account, the group conjugated APO2L/TRAIL in artificial lipid vesicles resembling natural exosomes, and used these artificial-exosomes as a treatment in a rabbit model of antigen-induced arthritis (AIA). These artificial-exosomes increased APO2L/TRAIL bioactivity and resulted in a more effective treatment of AIA compared with soluble, un-conjugated APO2L/TRAIL. Also, they reduced synovial hyperplasia and inflammation in rabbit knee joints [125]. Subsequently, they generated liposomes coated with bioactive APO2L/TRAIL to analyze their apoptosis-inducing ability on cell lines from hematological tumors. These liposomes (LUVs-APO2L/TRAIL) greatly improved APO2L/TRAIL activity [126].

Deng et al. prepared artificial-exosomes using lipids that were derived from intestinal, mucus-derived, exosome-like nanoparticles (IDENs). In brief, the liposomes were formed by extracted IDEN lipids which were hydrated, bath sonicated and probe sonicated. It was found that these artificial-exosomes possessed similar NK T-cell inhibitory activity to the original ones [127].

#### 4.2.2. Physical-Origin Extracellular Vesicle-Mimetics

Due to the low yield of extracellular vesicle isolation from cell media or other sources, extracellular vesicle-mimetics have also been composed by using other types of physical-origin media as starting material. Most of the physical-origin extracellular vesicle-mimetics studied to date are derived from whole cells. Some other hybrid type extracellular vesicle-mimetic systems are found in the literature, and will be mentioned separately.

Cell-derived vesicles or cellular vesicles (CVs) are a new and rapidly evolving class of biological drug delivery systems. They are inspired from extracellular vesicles, but represent a totally different type of endogenous nanocarriers. Cellular vesicles retain the surface characteristics of their parental cells, and are thus highly biocompatible with efficient intrinsic targeting ability (providing, of course, that they are of autologous origin); additionally, they don’t need any further surface functionalization. This offers a clear advantage over other (synthetic) drug delivery systems. Moreover, the fact that cellular vesicles are derived from natural sources yield a higher retention in the circulation and for a reduced clearance rate, increasing the circulation time of their therapeutic cargo in the body, without the fear of any stimulated systemic toxicity. These stable and long-circulating endogenous nanocarriers provide protection of the drug cargo from degradation, and increase drug delivery to targeted tissues [128,129]. Although the broader use of non-autologous cellular vesicles, as also exosomes, could not be envisioned for the same safety reasons mentioned in the case of (engineered)-exosomes, the substantially higher production yield of cellular vesicles could be the key difference that will perhaps enable the development of a road-map for rapid manufacturing of cellular vesicle-loaded drugs from autologous cells.

Cellular vesicles are obtained by subjecting cells to physical processes producing vesicles of nano-dimensions. The very small size of cellular vesicles (<200 nm) facilitates the cellular uptake of the delivered drug, due to the increased permeability and retention effect. However, the greater advantage of cellular vesicles is that they escape from innate immune recognition, avoiding the rapid degradation and clearance by the body’s immune defense systems. Indeed, synthetic nanoparticles are known to interact with the innate immune system, including the complement system, and these interactions with the immune system impose significant clinical limitations. Cellular vesicles, as natural carriers diminish complement activation responses and evade blood clearance by the immune system, enabling long circulation of their cargo, being thus ideal candidates of clinically approved nanocarriers.

Cellular vesicles are derived from the cell plasma membrane. They are composed of a lipid bilayer and have a final size between 50 and 200 nm. They are closed vesicular structures that incorporate many cellular contents from the parental cells, such as membrane proteins, intracellular proteins, and RNAs. Beside the different methods utilized for their production and isolation, cellular vesicles and extracellular vesicles present many similarities in terms of their size (nano-scale), morphology, key membrane proteins, and lipid composition [130]. As far as vesicle content is concerned, cryo-TEM and western blot analysis of isolated cellular vesicles showed that they include all the membrane proteins of their parental cells, and also preserve their topology, which is logical, since the lipid bilayer membrane of the cellular vesicles is directly derived from the plasma membrane of the cell. They also preserve the characteristic intracellular proteins of the original cells; therefore, cellular vesicles contain both membrane proteins from the parental cell membrane and intracellular proteins from the parental cells. In addition, RNA analysis with RT-PCR revealed that cellular vesicles enclose intracellular RNAs from parental cells. After RNA profiling of cellular vesicles, the presence of intracellular miRNA, rRNA, and tRNA was verified, indicating that cellular vesicles can be used as vehicles for RNA delivery.

Beside protein and RNA analysis of cellular vesicles, their lipid composition was also investigated in one study [128]. Specifically, it was demonstrated that cellular vesicles are more similar to exosomes than parental cells (U937 cells in this case) withregards to their major lipid constituents (phosphatidylcholine, phosphatidylethanolamine, sphingomyelin, and lysophosphatidylcholine). However, some differences were detected in the relative lipid amounts, which may imply different properties for each vesicle type. Interestingly, the lipidic profile of cellular vesicles has a strong resemblance to that of erythrocytes, a fact that could explain the high cellular uptake and long body circulation time of some cellular vesicle-types [128].

Cellular vesicles are produced by subjecting cells to physical processes (different procedures have been utilized), resulting in vesicles of nano-scale sizes. Many methods have been used and new methods are continuously emerging in the literature, including passing cells through extruders, through microchannels or through custom-made devices inside a centrifuge. The most commonly-used technique involves serial extrusions of the cells through polycarbonate membrane filters of decreasing pore sizes, usually starting form 10-μm, then 5-μm, 1-μm, and lower. The cellular vesicles are finally obtained by performing density gradient ultracentrifugation or size-exclusion column chromatography in order to realize sizes between 100 and 200 nm, which are required in order to take advantage of the enhanced cellular uptake and retention (EPR) effect and avoid premature clearance from the body [129,131].

Another cellular vesicle fabrication technique described in the literature is the cell-slicing system to generate vesicles from living cells. This method actually exploits the ability of the cell membrane lipid bilayer to self-assemble into vesicles. Specifically, living cells are flowing inside a microfluidic device and are serially sliced by 500 nm-thick silicon nitride blades while passing through microchannels lined with the blades. When cells are sliced, a free-standing piece of plasma membrane with an intact lipid bilayer structure forms a vesicle that contains the cellular contents of the parent cells [132].

The above described methods generate a high yield of cell-derived vesicles from living cells over a relatively short period of time. They are simple, rapid, and inexpensive methods that require only common laboratory equipment. Furthermore, when compared to extracellular vesicle isolation procedures, cellular vesicle fabrication methods are far superior, as they produce larger quantities of nanovesicles over a shorter time period. Specifically, the yield of isolated cell-derived vesicles is more than 100 times higher compared to that of extracellular vesicles [133,134]. Extracellular vesicle isolation and production methods usually require up to 4 days, whereas cellular vesicle production can be completed in a single day. The difference in processing time is mainly due to the time needed for the cells in culture to secrete enough extracellular vesicles for isolation. This step is unnecessary in CV-fabrication, as CVs are produced immediately after the cells in culture reach confluency [128].

Cellular vesicles, like extracellular vesicles, shuttle endogenous biological cargo such as mRNAs, RNAs, and proteins that can be efficiently taken up by target tissues. Moreover, some recent reports showed that CVs can efficiently encapsulate and deliver exogenous biomolecules to targeted sites [132,133]. Specifically, Yoon and colleagues demonstrated (by using the cell-slicing system) that during self-assembly, the plasma membrane fragments envelop exogenous materials from the buffer solution. About 30% of material can be encapsulated in cellular vesicles, and the generated vesicles can efficiently deliver the exogenous cargo across the plasma membrane of recipient cells [129,132]. On the other hand, Jang et al. [133] showed that by subjecting cells of different origin to serial extrusions through filters with decreasing pore sizes after the cells had been loaded with chemotherapeutic agents, high quantities of drug-loaded cellular vesicles are generated.

Recent reports present cellular vesicles as viable nanocarriers for drugs in the treatment of various diseases. For cancer treatment, cell-derived vesicles seems to have an advantage as they achieve passive targeting via enhanced permeability and retention effect on leaky vasculature of tumors. Indeed, CVs were demonstrated to successfully deliver different chemotherapeutic substances, such as doxorubicin, at high amounts to tumor tissues and significantly reduced tumor growth without the adverse effects observed with equipotent free drug [129,132]. In another study, researchers utilized cellular carriers in liver regeneration. Cellular vesicles were produced by serial extrusions of primary hepatocytes and were then injected intravenously in mice. It was clearly shown that the vesicles contained the protein content of the parental cells, and that they efficiently promoted hepatocyte proliferation in vitro, and liver regeneration in vivo [131]. Strategies inspired by this study could lead to the usage of cell-derived vesicles in tissue repair and regeneration, as well as in drug delivery.

#### 4.2.3. Other Types of Extracellular Vesicle-Mimetic Systems

Several other types of extracellular vesicle-mimetic systems can be found in the literature, which are actually hybrid systems aiming to combine the advantages of exosomes with those of other systems. Actually, the hybrid systems produced after fusion between liposomes and exosomes [54] (mentioned above) can be included in this category instead of the category of engineered-exosomes.

As an example of other hybrid systems (that will not be discussed further herein), we mention a previous case where non-enveloped viruses were associated with extracellular vesicles to form virus-extracellular vesicle-hybrid particles that combine the respective advantages of extracellular vesicles (low immunogenicity, targeting) and viruses (stable gene expression). Indeed, the hepatitis A virus released by cells was cloaked in host-derived membranes that resemble exosomes, and this hybrid system was found to be fully infectious [135].

## 5. Methods of Preparation and Engineering of Engineered Exosomes and Exosome-Mimetics

In the next part, we will focus on the methodologies used to date for drug loading and the surface modification of various types of extracellular vesicles. The same methodologies to load drugs also apply for extracellular vesicle-mimetics.

### 5.1. Drug Loading Methodologies

**Scheme 4:**
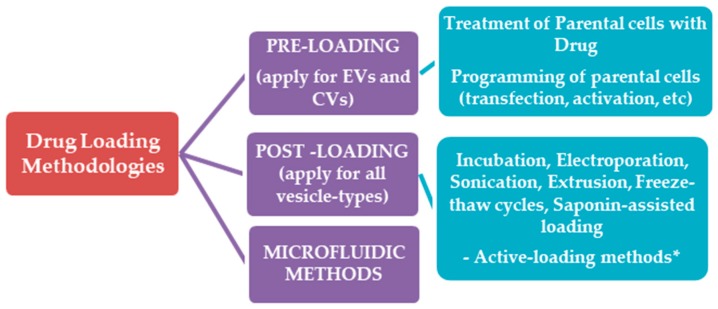
Categories of methods used for loading drugs into EXs and EX-like vesicles.

The drug loading methodologies applied to date in the case of exosomes, and their physical mimetics (Cellular Vesicles), are categorized in two main groups, the pre-loading methods and the post-loading methods. The recently-developed microfluidic approaches are separately discussed (Scheme 4).

In pre-loading methods, the drug is initially produced or loaded in the parental cells, and thus, the extracellular vesicles or cellular vesicles isolated or produced by them are already pre-loaded with the desired drug. Such methodologies are particularly useful when oligonucleotides or proteins are to be loaded in the vesicles, in which case the cells can be programmed to produce “a-la-carte” extracellular vesicles or cellular vesicles (after applying particular cell engineering techniques).

In post-loading methods, the drug is loaded in the extracellular vesicles after their isolation. In recent years, scientists have been trying to apply high yield drug loading methods that have been used for liposome engineering, and in some cases, the loading efficiencies acquired are significantly improved, when compared to the simple methods used in earlier reports (such as electroporation and simple incubation). Nevertheless, we have not been able to detect any reports about the potential to use special loading methods that have been very successful for efficient loading of amphiphilic drugs into liposomes, such as the “active or remote” loading methods for amphiphilic drugs, such as Doxorubicin etc. [136], which can be developed as a “one-step” manufacturing methods on a microfluidic setup, as recently proven [137]. The only relevant report found was that of Zhang et al., [138] that tested the remote loading method in red blood cell (RBC) ghosts that were reconstructed into vesicles. It was found that the membranes needed to be enriched with cholesterol in order to retain a fluorescent dye, and up to 10% cholesterol was tested, while the fluorescence signal reached a plateau after 5% cholesterol. The 5% cholesterol-enriched vesicles were remotely loaded using an ammonium sulfate/PBS gradient; the maximum loading reported was only 10% for DOX, and 40% for vancomycin. The authors explain the difference by the higher MW of vancomycin, implying that the vesicles were still not stable enough to retain low-molecular weight molecules. The fact that DOX was shown to be precipitated in the vesicles does not comply with this explanation. Furthermore, the group did not try to optimize the method, by perhaps adding more cholesterol in the vesicles, or by other means. More recently, the same group tested remote loading for platelet vesicles, using identical protocols for vesicle engineering and loading, and testing the same drugs; they found that 5% cholesterol-enriched platelets could be loaded with up to 11% DOX and 33% Vancomycin, giving the same explanation for the difference, while, again, DOX was verified to be entrapped in the vesicles in a crystalline form [139]. Thereby, remote loading in such exosome-like vesicle types could be further exploited for optimization.

In the next part, we present the most commonly-applied methods to date and their results, and also discuss potential future challenges. In order to avoid misunderstanding, we should mention at this point that many previously-applied exosome-loading methodologies, which are known in the liposome field as “passive” loading methods, since the drug is entrapped in the liposomes passively due to a concentration gradient (incubation of vesicles with drug solutions) or because the liposomes are formed in the drug solution (thin film method, sonication, extrusion, etc.), have been characterized in several previous review articles as “active-loading methods”. We clarify here that in liposome technology, we use the term “active loading” or “remote loading” only for techniques such as the ion or pH trans-membrane gradient methods, as mentioned above [136,137].

#### 5.1.1. Pre-Loading Methods

##### Treatment of Parental Cells with Drugs

In this type of method, the parental cells are treated with a drug, and the cells subsequently secrete drug-pre-loaded exosomes or extracellular vesicles. Although it is impossible to control the loading efficiency with this type of methods, several studies, which are summarized below, have been conducted using such techniques, owing to their simplicity.

Pascucci et al. encapsulated paclitaxel (PTX) into murine SR4987 mesenchymal stroma cells after culturing the cells with a low dose of PTX for 24 h, and subsequently, washing and reseeding them with fresh medium. After 48 h, PTX-loaded-exosomess primed with high dose of PTX were collected. The PTX-loaded exosomes showed strong anti-proliferative activity against CFPAC-1 human pancreatic cells, as compared with control exosomes which were isolated from untreated cells [104].

Lv et al. isolated extracellular vesicles from resistant hepatocellular carcinoma cells (HepG2) cells treated with different drugs (paclitaxel, carboplatin, etoposide and irinotecan hydrochloride). After exposure to the anticancer drugs, membrane microvesicles confirmed by TEM and Western Blot to be exosomes, were actively released by the cells. The later exosomes differed in their ability to present heat shock proteins (HSPs) on the cell surface. Their size ranged between 30 and 100 nm with the majority of the vesicles being around 70–90 nm [106].

Another approach is the one developed by Lee et al. a method of liposome-mediated extracellular vesicle engineering for anticancer drug-loading. Synthetic fusogenic liposomes which were loaded with hydrophobic drugs were efficiently incorporated into the host cell membrane and the drugs were thus subsequently loaded into the membranes of the cell-secreted extracellular vesicles [140]. The same group used this method to introduce lipid-azides in the membrane of extracellular vesicles which were further conjugated with targeting moieties and loaded with Paclitaxel (PTX) and tirapazamine (TPZ) [141].

##### Parental Cell Engineering

In the last decade, an increasing number of reports in the literature describe methods for successful loading of therapeutic cargo in exosomes, by modulation of the exosome-producing parental cells. The most common cell engineering techniques involve transfection or activation of parental cells.

Transfection is the most widely-used and efficient method for the loading of therapeutic proteins or oligonucleotides into exosomes. RNA (miRNA, siRNA, and mRNA) and protein sequences are easily transfected as synthetic oligonucleotides or expressed from a plasmid backbone. Cell transfections are carried out by the calcium phosphate method or by commercially-available lipid transfection reagents such as Lipofectamine, or others. With such methods, biological cargos can be packaged into exosomes to promote or silence gene expression or regulate transcription in recipient cells. Studies have shown that transfection can also be used to overexpress a specific protein on the surface membrane of the exosomes, or the proteins could be packaged into the exosome lumen. Indeed, in addition to modifying exosome membranes through genetic engineering of their parent cell, the therapeutic cargo of exosomes may also be manipulated by altering various aspects of their regulated biogenesis.

miRNAs were successfully introduced into exosomes in several studies by using miRNA expression vectors. Akao et al. showed that transfecting modified miR-143 in THP-1 macrophage cells led to the successful loading of the modified miRNA into the exosomes. This work exhibited that when overexpressing a miRNA into parental cells, this effectively leads to the passive load of miRNA into exosomes [142]. Ohno et al. exhibited that the miRNA-loaded exosomes could also be targeted effectively to recipient cells after engineering their surface with targeting peptides, and demonstrated that intravenously injected exosomes accumulated in the tumor site and reduced the tumor growth [143].

Interestingly, cell activation has been used as an approach for loading functional cargo into exosomes. While it is not the most appropriate method for exosome loading, it shed light on some aspects of the physiology and function of exosomes. Cell activation as a methodology for exosome loading was, however, only studied a few times. Zhang et al. demonstrated that when the human monocytic cell line THP-1 was stimulated with different inflammatory stimulants, increased levels of miR-150 in the resulting exosomes were shown, with subsequent increased endothelial cell migration [144]. Furthermore, Xin et al. showed that brain extracts from rats undergoing middle cerebral artery occlusion exhibited increased expression of miR-133b in multipotent mesenchymal stromal cell (MSC)-derived exosomes after co-culture of MSCs with neurons and astrocytes [145].

Another strategy for loading exosomes with nucleic acids exploits viral packaging mechanisms. During the production of adeno-associated virus (AAV) vectors, a fraction of the vectors was associated with exosomes, and outperformed conventionally-purified AAV vectors in transduction efficiency [146].

#### 5.1.2. Post-Loading Methods

In this section, the post-loading methods which have been used to date for drug loading into engineered- exosomes of artificial-exosomes are described, starting from the simplest methods and continuing with the most complicated ones (usually applied in liposome technology).

##### Incubation with Drug

The simplest way to incorporate any cargo into exosomes is their co-incubation, simply by mixing the isolated vesicles with the drug. The driving force for the loading is the different concentration of the drug in and out of the vesicle membrane. Hydrophobic drugs interact with the vesicles lipid layers, and the drugs diffuse into the exosome cavity along the concentration gradient. The low loading capacity is considered as the main drawback of this method. The loading efficiency of co-incubation method depends on the lipophilic properties of the drug, as well as the concentration gradient [92]. Some examples of drug loading into exosomes by their incubation with the drug of interest are presented below:

Sun et al. incubated curcumin with exosomes in PBS at 22 °C for 5 min and the exosomes were subsequently purified [92]. Curcumin was thus self-assembled into the lipid bilayer of exosomes via hydroscopic interactions, resulting in increased drug stability. Using the same method, Zhuang et al. encapsulated both curcumin (Exo-Cur) and a signal transducer and activator of transcription 3 (Stat3) inhibitor (Exo-JSI124), by mixing curcumin or JSI124 with EL-4 cell derived exosomes in PBS. After incubation at 22 °C for 5 min, drug-loaded- exosomes (Exo-cur or Exo-JSI124) were subsequently isolated [107]. Didiot et al. developed a robust, efficient, and highly-reproducible method for loading therapeutic RNA into exosomes upon co-incubation of hydrophobically modified small interfering RNAs (hsiRNAs) and exosomes, without affecting either the vesicle size or their integrity [116]. Fuhrmann et al. reported the loading of porphyrins of different hydrophobicities intro exosomes by incubation with the drug at RT for 10 min [111], while Haney et al. successfully loaded the enzyme catalase (a tetramer protein of 250 kDa) into RAW264.7 cell-extracted exosomes in PBS at RT for 18 h [108].

##### Electroporation

By this technique, an electrical field disturbs the phospholipid bilayer of vesicles (such as extracellular vesicles or exosomes or cells), creating small pores in their membrane, and thus allowing the passage of the drug into the vesicles. The integrity of the vesicle membrane is then recovered, resulting in the formation of drug-loaded vesicles.

Electroporation has been used for loading siRNA into exosomes [112]. The idea is that siRNA molecules, which are relatively large and cannot diffuse into exosomes spontaneously due to their hydrophilic nature, could be assisted by electroporation in order to diffuse into exosomes. Wahlgren et al. proved that electroporation leads to superior loading of siRNA over chemical transfection [113,115]. In more detail, in order to overcome the toxicity related with the use of cationic liposomes for the delivery of nucleic acids (due to electrostatic complexation of the negatively charged nucleic acids with cationic lipids), this group used exosomes which could prevent the electrostatic complexation between nucleic acids and cationic lipids, due to the negative surface charge of exosomes. For this, two different strategies, electroporation and chemical transfection, were applied to introduce a double-stranded siRNA into the exosomes. For the chemical transfection, a siRNA against MAPK1 was mixed with the transfection reagent to allow the formation of transfection complexes (siRNA embedded in lipid micelles). The complex was then incubated with the exosomes and vesicles were subsequently purified. However, since it was impossible to separate the exosomes from the complex, chemical transfection was found to be inapplicable as an exosome-loading method. On the other hand, electroporation was found to result in efficient exogenous siRNA loading into the same exosomes [113]. Nevertheless, the use of electroporation as a method to load siRNA into exosomes has been criticized. In fact, Kooijmans et al. stated that electroporation disrupts exosome integrity, and is “far less efficient than previously described”. They showed that electroporation of exosomes in presence of siRNA is accompanied by extensive siRNA aggregation, which may cause overestimation of the amount of siRNA actually loaded into the exosomes [147]. In an effort to overcome this issue, Johnsen et al. reported that when electroporation is carried out in an optimized buffer such as trehalose disaccharide, the buffer can aid the structural integrity and inhibit the aggregation of exosomes extracted from adipose-derived stem cells (ASCs). In more detail, when exosomes were resuspended in electroporation buffers, such as cytomix electroporation buffer or trehalose pulse medium, and electroporation was performed at 400 V and 125 µF, morphological changes and aggregation of exosomes were observed; conversely, when the trehalose-containing buffer system was used during electroporation, the structural integrity of the exosomes was preserved [148]. In the same direction, Lamichhane et al. used electroporation to load small RNAs (siRNA, miRNA or single stranded DNA (ssDNA)) into exosomes isolated from two different cell lines, HEK293T and MCF-7, by alleviating nucleic acid aggregation. They mixed exosomes with nucleic acids in electroporation buffer, and electroporation was carried out at 400 V and 125 μF with two pulses. All samples were purified and subsequently transferred into tubes, where EDTA was added to alleviate nucleic acid aggregation. Samples were then incubated at room temperature for 15 min and centrifuged at 5000× *g* at 4 °C for 5 min to remove buffer and unincorporated cargo. [114].

Despite oligonucleotides, electroporation has been successively used to encapsulate small hydrophilic drugs into exosomes. Fuhrmann et al. encapsulated TMP (5,10,15,20-tetrakis (1-methyl-4-pyridinio) porphyrin tetra (*p*-toluenesulfonate)) which is used for its photodynamic effects. Electroporation was performed at 200 Ω, 500 μF, 200 mV and a pulse time of 20–30 ms [111]. Other examples of drug loading into exosomes by electroporation include DOX loading into mouse immature dendritic cells (engineered to express Lamp2b (an exosomal membrane protein) fused to αv integrin-specific iRGD peptide (CRGDKGPDC)) derived exosomes. The purified exosomes were efficiently loaded with DOX via electroporation [109].

Nakase and Futaki used the electroporation technique to encapsulate a dextran macromolecule (70 kDa) into exosomes which were isolated from CD63-GFP-HeLa cells. The exosomes combined a cationic lipid (Lipofectamine) and a pH-sensitive fusogenic peptide (GALA) for the fusion of endosomal and exosomal membranes inside cells [110].

##### Sonication

In this method, exosomes derived from donor cells are mixed with drugs and subsequently sonicated by a probe sonicator which allows the drug to flow into the exosomes due to the sonication-induced deformation of their membrane. In principle, reformation of exosomal membranes under sonication may enable the passage of drugs across relatively tight lipid bilayers [105]. Some examples of drug loading with sonication are presented below:

Kim et al. tested sonication as a method to load paclitaxel (PTX) into macrophage released exosomes; the method resulted in high loading efficiency and sustained drug release, while it did not significantly affect the protein and/or the lipid contents of the exosomes [105].

Haney et al. effectively loaded catalase into exosomes. The catalase- exosome mixture was sonicated (500 V, 2 kHz, 20% power, 6 cycles by 4 s pulse/2 s pause), cooled down on ice for 2 min, and then sonicated again [108].

Sonication was also used as a method to load small RNAs (siRNA and miRNA) into exosomes (and microvesicles). However, the application of exosomes for therapeutic RNA delivery may be limited by such loading approaches that may induce cargo aggregation or degradation. Lamichhane et al. loaded functional small RNAs (siRNA, miRNA or single stranded DNA (ssDNA)) into exosomes isolated from two different cell lines, HEK293T and MCF-7 by sonication. For this, the nucleic acids were incubated with the exosomes at RT for 30 min, followed by sonication in a bath sonicator at 35 kHz for 30 s. Mixtures were then placed on ice for 1 min and sonicated again for the same time period. These conditions led to minimal detectable aggregation [114,149], probably due to the mild sonication conditions applied.

##### Extrusion

In this method, exosomes are mixed with a drug, and the mixture is loaded into a syringe-based lipid extruder and extruded through membranes with 100–400 nm porous size, at controlled temperature. During the extrusion, the exosome membrane is disrupted and vigorously mixed with the drug, resulting in drug loading into the exosomes.

Fuhrmann et al. loaded porphyrins of different hydrophobicities into exosomes by extrusion. The procedure was conducted at 42 °C using a syringe-based, hand-held mini-extruder equipped with a heating block, and polycarbonate membranes of 400 nm pore size. Each sample was extruded 31 times. It was found that the extrusion method resulted in alteration of the zeta potential of the vesicles, possibly due to modulation of the constitution of the lipid membrane of original exosomes. The extruded exosomes were demonstrated to cause cytotoxicity, whereas exosomes loaded with the same porphyrin but prepared by other methods did not show significant cytotoxicity [111].

Catalase has also been loaded into RAW264.7 macrophage-derived exosomes by extrusion. For this, the catalase- exosome mixture was efficiently extruded (×10 times) through 200 nm-pore diameter filters [108].

##### Freeze/Thaw Cycle Method

In this method, drugs are mixed and incubated with exosomes at RT, and the mixture is subsequently frozen at −80 °C or in liquid nitrogen, and re-thawed at RT. This process is repeated for at least 3 cycles to ensure drug encapsulation [54]. However, by this method exosomes, may aggregate, while the drug loading efficiency is generally lower than that of sonication or extrusion.

The freeze-thaw method has been used for catalase incorporation into exosomes. The catalase solution was mixed with the exosomes, incubated for 30 min, then rapidly freezed at −80 °C, and thawed at RT. The freeze-thaw cycle was repeated three times [108].

Sato et al. proposed a new approach to prepare engineered hybrid exosomes by fusing the membranes of exosomes with those of liposomes, using a freeze-thaw method for this. Raw 264.7 cell-derived exosomes were mixed with fluorescently-labelled liposomes composed of phospholipids, pegylated-phospholipids, and fluorescently labeled phospholipids. The mixtures were frozen in liquid nitrogen and thawed at RT for 15 min. To further examine the applicability of the membrane fusion technique, they investigated the fusion behavior between exosomes bearing a specific membrane protein (HER2) and liposomes. HER2 and phosphorylated HER2 were detected in the exosome-liposome mixtures, indicating that exosome-liposome hybrids carrying specific proteins can be obtained by freeze-thaw methods. Cellular uptake studies performed using the hybrid exosomes revealed that the interactions between the developed exosomes and cells could be modified by changing the lipid composition, and consequently, also the properties of liposomes [54].

##### Saponin-Assisted Loading

Saponin is named from the Latin “sapo” which meaning “soap”; it is a surfactant molecule that, upon incubation with exosomes, generates pores in their membrane through interactions with cholesterol, leading to increased exosomes-membrane permeability. Saponins can be composed of one to three straight or branched sugar chains, and are most often composed of d-glucose, l-rhamnose, d-galactose, d-glucuronic acid, l-arabinose, d-xylose, or d-fucose. The sugar chain can contain from one to several monosaccharide residues, and is usually attached at C-3 [150]. The surface activity, as well as some other biological functions of saponins (including their haemolytic activity), are attributed to their characteristic structural features and their amphiphilic nature which is are the results of the presence of a hydrophilic sugar moiety and a hydrophobic genin (called sapogenin) [150].

Haney et al. loaded catalase into exosomes ex vivo using different methods. For the loading by the saponin-assisted method, a mixture of catalase and exosomes (from Raw 264.7 macrophages) was supplemented with 0.2% saponin and placed on a shaker for 20 min at RT. This method resulted in high loading efficiency, sustained release, and catalase preservation against proteases degradation. Moreover, although saponin is a surface-active agent, it did not degrade catalase, whose activity was preserved [108].

Saponin can also assist in loading other hydrophilic molecules into exosomes. For saponin-assisted drug loading of hydrophilic porphyrins, exosomes and the drug were incubated with 0.1 mg/mL saponin at RT for 10 min. High drug loading was assessed by this method [111].

However, there are concerns regarding the in vivo hemolytic activity of saponin. Haemolysis of red blood cells seems to result from the ability of saponin to form complexes with the cholesterol of the cell membrane, leading to the formation of pores, cell membrane permeabilization, and alterations in the negatively-charged carbohydrate portions on the cell surface; however, the exact mechanism of the haemolytic activity of saponins is not clear. In any case, when used as a method to assist drug-loading into exosomes, the concentration of saponin should be kept to a minimum, and the exosomes should be washed immediately after incubation with saponin [150].

#### 5.1.3. Comparison of Different Loading Methods

Table 1 summarizes the advantages/disadvantages of the aforementioned pre- and post-loading methods. In some reports, several methods were used for loading drugs into the same exosomes, and thus, the methods could be accurately compared:

Haney et al. used various methods in order to encapsulate catalase (a 240 kDa protein) into exosomes (exoCAT) for Parkinson’s disease therapy. In fact they used: (a) incubation at RT, (b) saponin-assisted loading, (c) freeze-thaw cycles, and (d) sonication and extrusion. In all cases, the size of the obtained catalase-loaded exosomes was in the range between 100 nm and 200 nm. Sonication and extrusion or saponin-assisted loading resulted in stable exosomes with high loading efficiency, sustained release, preservation against proteases degradation, and proven (in vitro and in vivo) capability for targeted delivery [108].

Fuhrmann et al. applied several techniques for the loading of porphyrins with different hydrophobicities into exosomes. Specifically, they used: (a) incubation of drug with exosomes at RT, (b) electroporation, (c) saponin-assisted loading, (d) extrusion, and (e) Hypotonic dialysis. Details for the exosome preparation according to methods (a)–(d) have already been discussed above. Hypotonic dialysis (method e) was performed by transferring the exosome-drug mixture into dialysis membranes (cellulose ester, MW cut-off of 100–500 Da) which were placed in 10 mM PBS (pH 7.4) and stirred at RT for 4 h. The loading efficiencies acquired were dramatically higher when co-incubation with 0.01% (*w*/*v*) saponin or hypotonic dialysis was used. For both of the latter two methods, the loading of the drugs was up to 11-fold higher compared with the efficiencies acquired by the other methods tested (incubation, electroporation and extrusion) [108].

Kim et al. tested different methods to load PTX into macrophage-derived exosomes. The methods investigated were: (a) incubation at RT, (b) electroporation, and (c) sonication. The amount of PTX loaded into exosomes was found to increase as follows: incubation at RT < electroporation << sonication. It was also found that reformation of the exosomal membrane upon sonication resulted in high loading efficiency and sustained drug release. The authors also proved that the mild sonication utilized did not significantly affect the protein and/or lipid content of exosomes [105].

Goh et al. used 4 different methods to load doxorubicin (DOX) into cellular vesicles: (a) incubation at 37 °C for 5 min, (b) incubation with 0.2% saponin for 5 min, (c) incubation for 24 h at RT, and (d) freeze-thaw cycles (3 cycles). DOX loading by addition of 0.2% saponin resulted in the highest loading efficiency, but also in increased cellular vesicle size. When incubation at RT for 24 h was used, low loading efficiency and a large increase in size were realized, suggesting that aggregation of cellular vesicles occurred with DOX acting as an intercalating agent. Incubation of DOX with cellular vesicles at 37 °C seemed to yield a balance between size and loading, and 40% of the drug was released after 36 h (in vitro). A much slower release (20% of the loaded-DOX after 36 h) was observed in the case of the saponin-assisted DOX-loaded cellular vesicles [129].

Lamichhane et al. reported the use of sonication and electroporation as methods to load siRNA into exosomes. They suggested that sonication may be a suitable alternative to electroporation for small nucleic acid loading in extracellular vesicless. They also reported that the use of sonication may be superior to electroporation with respect to loading efficiency and siRNA aggregation [114].

### 5.2. Surface Modification Methods

The proteins expressed on the surface of exosomes compose an integral variable in exosome biodistribution and cell-targeting capabilities. Modification of surface proteins aims to improve the targeting efficiency of exosomes to tissues and cells types of interest. Exosome components can be engineered at the cellular level, or they can be modified after their isolation [151].

At the cellular level, one approach is the use of cellular transgene expression to develop a modified exosome membrane protein with specific signaling or homing properties. This can be done by inserting the coding sequence of the ligand of interest into frame to the coding sequences of the signal peptide and the amino-terminus of the peptide of the specific membrane protein. By using a gene transfer vector, such as retroviral or lentiviral vectors, this fusion cassette is expressed in parental cells, and consequently, the transduced cells generated exosomes expressing the peptide of interest on their surface. The most commonly-modified transmembrane proteins include tetraspanins (CD63, CD9, CD81), lysosome-associated membrane glycoprotein 2b (Lamp-2b), glycosyl-phosphatidyl-inositol (GPI), platelet-derived growth-factor receptors (PDGFRs), and lactadhein (C1C2 domain) [152].

Alvarez-Erviti et al. [112] fused the rabies viral glycoprotein (RVG) with Lamp-2b to specifically deliver exosomes to neurons and glia. Targeted exosomes successfully crossed the blood-brain barrier and delivered their silencing RNA cargo, resulting in BACE1 knockdown. In a similar way, Tian et al. managed to modify immature dendritic cells to express Lamp2b fused to αv integrin-specific iRGD peptide [109]. Another study by Liu et al. showed that engineering the membrane surface of exosomes to express the RVG peptide effectively delivers opioid receptor mu siRNA into the brain [153].

Several examples of methods were used to modify the surface of exosomes after their isolation. Nevertheless, the reaction conditions for exosome surface modification/functionalization must be strictly specified in order to avoid exosome disruption and aggregation due to inappropriate temperature, pressure, and/or osmotic stress. Among the methods that are available for cross-linking reactions, the copper-catalyzed azide-alkyne cycloaddition (CCAAC), known as click chemistry, has several advantages. Click reaction is achieved by the reaction of an alkyne chemical group and an azide chemical group to form a triazole linkage. The reaction works both in organic and aqueous media, making it ideal for exosome (or liposome) surface modification, while it is also rapid, efficient, and offers chemo-selectivity over the conjugation site, compared to other traditional cross-linking reactions [154,155,156]. Some examples of click chemistry modifications of exosomes are summarized below.

Smyth et al. attached fluorescent molecules to the surface of mouse 4T1 breast cancer exosomes by efficiently functionalizing exosomes with terminal alkyne groups. In order to do this, the amine groups of the exosomal proteins (or the exosomal membrane lipid phosphatidylethanolamine) were cross-linked with the carboxyl group of 4-pentynoic acid using carbodiimide activation. The alkyne-functionalized extracellular vesicles were conjugated with azide-fluor 545 using click chemistry [156]. No differences in morphological and functional properties were found, suggesting that modification by click chemistry does not alter exosome characteristics, allowing the incorporation of exogenous molecules on the surface of exosomes. In order to investigate the extent of exosomal protein modification with alkyne groups, they used liposomes with surface alkyne groups of a similar size and concentration to exosomes, and estimated that approximately 1.5 alkyne groups were present for every 150 kDa of exosomal protein.

Based on previous reports of Lakrishnan et al., Lee et al. used membrane fusogenic liposomes (MFLs) to insert azide-lipids in the plasma membrane [157]. Lipid-azide was efficiently delivered to the plasma membrane via MFLs and subsequently loaded into the membrane of exosomes that were formed. More specifically, cancer cells were treated with liposomes bearing lipid-azide (azide-MFLs), and the exosomes produced from the cells showed significantly marked fluorescence when reacted with DBCO-carboxyrhodamine, verifying that exosomes containing azide-lipids through liposome-based cellular engineering could be decorated easily with various functional moieties by using copper-free click chemistry. Azide group-bearing-Exosomes were further decorated with targeting moieties in order to allow their specific delivery to cancer cells. Exosomes were also packaged with chemotherapeutics through liposome-based cellular engineering. Paclitaxel (PTX) (a hydrophobic anti-cancer drug) and tirapazamine (TPZ) (a hydrophilic agent that becomes cytotoxic selectively in hypoxic conditions) were loaded in exosomes and delivered to target cells [141].

Besides covalent bonding on the exosome-surface, some non-covalent strategies have additionally been used in order to provide stable modification of the exosome surface. In general, multivalent electrostatic interactions and receptor-ligand binding are commonly used for the surface modification of exosomes.

Multivalent electrostatic approaches typically involve a highly cationic species adhering to negatively-charged functional groups present on biological membranes. Nakase and Futaki used electrostatic interactions to bind cationic lipids to the surface of exosomes. Such interactions produced exosomes with a positively-charged surface potential that enhances binding and uptake into recipient cells. [110]. However, there are concerns that certain cationic nanomaterials can cause cytotoxicity [158]. Moreover, cationic nanomaterials are typically taken up into the cell via endocytosis, leading to lysosomal degradation and low exosome loading efficiency [110].

Receptor-ligand binding was achieved by Qi et al., who developed a dual-functional exosome-based superparamagnetic nanoparticle cluster as a targeted drug delivery vehicle for cancer therapy. They efficiently conjugated transferrin-superparamagnetic nanoparticles to the surface of blood derived exosomes by targeting the tranferrin receptors on the exosome surface [159].

An alternate approach to attach proteins on exosomes relies on the trafficking of oligomeric membrane-anchored proteins to exosomes. The exogenous protein is fused with two domains that mediate aggregation and membrane localization, e.g., by adding a myristoyl- moiety. This approach was validated using a GFP reporter protein [160]. However, the potential interference of the protein oligomer formation with the functionality of the proteins in the target cells remains of concern [161].

Very recently, a holistic approach was proposed as a method to facilitate the isolation together with loading and surface modification (for improved targeting efficiency) of exosomes. Based on the fact that loading and targeting of patient-derived exosomes should be carried out without altering the exosome surface, Gao et al. demonstrated that a phage display identified a peptide (CP05) may enable targeting, cargo loading, and isolation/purification of diverse-origin- exosomes (including patient-derived ones), through binding to CD63—an exosomal surface protein. Indeed, the systemic administration of exosomes which were loaded with CP05-modified, dystrophin splice-correcting phosphorodiamidate morpholino oligomer (EXOPMO), resulted in an 18-fold increased dystrophin protein in dystrophin-deficient mdx mice compared to the control. Loading CP05-muscle-targeting peptide on the previous exosomes further increased dystrophin expression in muscle with functional improvement and without any detectable toxicity. Thus Gao et al. demonstrated that an exosomal anchor peptide can be used as a tool for holistic EX engineering, probing gene function in vivo, and targeted therapeutic drug delivery [162].

### 5.3. Microfluidic Methods for Engineering of Exosomes and Exosome-Mimetics

As mentioned above, microfluidic technologies and lab-on-chip methods have been recently applied in some cases for (i) the isolation/ purification of exosomes form cell media or other sources/biological fluids [86,87,88,89] and (ii) for the preparation of cellular vesicles from whole cells [132].

It is also known today that microfluidic methods are applied by many labs in the preparation of liposomes and other types of nanoparticles which are used for drug delivery. In fact, it has been proven that even ligand-targeted liposomes can indeed be prepared in such “one step”, fully-automated, controllable, and scalable microfluidic systems [7,163]. Such technologies, being scalable, easily automated, and highly reliable, can indeed assist in the development of roadmaps for manufacturing of artificial exosome-mimetics, accelerating their translation into pharmaceutical products. Whether such methods can also assist efforts to increase the yield of exosome isolation remains to be explored.

## 6. Potential Clinical Applications of EXs and EXs-Mimetics

### 6.1. Current Status

To date, most of the reports related with the use of exosome-like vesicles for drug delivery concern the use of exosomes derived from cells, such as cancer cells, dendritic cells, MSC, from biological fluids [27,29,53,54,55,56,57,58,59,60,61,62,63,64], or from other types of sources such as milk of fruits [69,70,72]. Cellular vesicles and totally artificial exosomes (or exosome-inspired liposomes) have been studied in a limited number of cases as drug delivery systems, since this specific research topic has been initiated very recently. Most of the studies are actually early preclinical proof-of-concept studies, to prove: (i) the possibility of exosome-like vesicles being loaded with sufficient amount of drugs; (ii) their capability to retain the drug under in vivo simulating conditions; and (iii) their potential to deliver the drugs in a functional state to the target cells of interest, at higher amounts compared to the free drug or other types of nanocarriers. In several cases, in vivo studies have also been carried out in appropriate disease models, verifying the in vitro findings.

The pre-clinical studies performed to date concerning the usage of exosome-like vesicles for drug delivery aim to treat several potential diseases such as different types of cancers [164,165,166], cardiovascular diseases [167,168], Parkinson’s and Alzheimer’s disease [169], as well as other neurodegenerative diseases [170,171], musculoskeletal diseases [172], kidney and diabetes-related pathologies [173], and others.

The potential of such exosome-like vesicles to overcome the BBB has also been under investigation [17,174]. In addition to some examples mentioned before, in which exosomes were engineered to attach brain homing peptides or antibodies that target the brain endothelium in order to enhance their brain targeting efficiency, in a recent study, it was demonstrated that there is no need for such modification to penetrate the BBB. Indeed, it was proposed that naïve macrophage exosomes can utilize the integrin lymphocyte function-associated antigen 1 (LFA-1) and intercellular adhesion molecule 1 (ICAM-1), and additionally, the carbohydrate-binding C-type lectin receptors, to interact with the BBB microvessel endothelial cells. It was demonstrated in vivo that naïve macrophage-exosomes cross the BBB and deliver a cargo protein, the brain derived neurotrophic factor (BDNF), to the brain following i.v. injection. Furthermore, the delivery is enhanced in the presence of brain inflammation, a condition which is often present in CNS diseases. Thereby, naïve macrophage-derived exosomes can function as nanocarriers for brain delivery of therapeutic proteins to treat CNS diseases [175].

In terms of the current clinical studies involving exosome-like materials, a search in the US-NIH clinical trial database (https://clinicaltrials.gov/) using the keyword “exosomes” resulted in 98 clinical studies, most of which were related with biomarker identification and diagnosis/prognosis or therapy of various types of cancers. Ten of the therapeutic studies are listed in Table 2 as examples of cases that are related with their potential use for the delivery of drugs. From these examples, only one is directly involved with the application of engineered exosomes as a DDS, specifically, the “Study Investigating the Ability of Plant Exosomes to Deliver Curcumin to Normal and Colon Cancer Tissue”, which involves the use of plant-derived exosomes for the delivery of curcumin (Table 2). The background of this study is related with the work from the James Graham Brown Cancer Center that suggests the use exosomes as a delivery vehicle to overcome all the major obstacles of using curcumin as an anti-inflammatory agent, providing increased stability, solubility, and bioavailability of curcumin [176]. The work was further extended to define the resource that can supply a large quantity of exosomes with a maximum binding capacity of curcumin, and emerging data indicated that fruits release exosome-like particles, that could strongly bind to many hydrophobic drugs including curcumin, and are taken up by the intestinal cells as well as the immune cells in the intestine. These results suggest that these fruit-derived exosomes are potentially used as a delivery vehicle to treat intestinal disorders. Moreover, both fruit exosomes and curcumin should not generate any side-effects, since they are consumed by humans daily. In this clinical trial, the effect of exosomally-delivered curcumin on the immune modulation, cellular metabolism, and phospholipid profile of normal and malignant colon cells in subjects who are undergoing surgery for newly-diagnosed colon cancer will be characterized. In selected subjects, the effect of exosomally-delivered curcumin on the production of cytokines, the changes of immune cells, and glucose metabolism by administration of 13C-glucose prior to surgical resection will also be characterized. Although the study is active, it has not yet started recruiting; therefore, no details or results are listed.

From the other studies listed in Table 2, the most relevant to this review include:

A phase I trial entitled “iExosomes in Treating Participants With Metastatic Pancreas Cancer With KrasG12D Mutation”, which will study the best dose and side effects of mesenchymal stromal cell-derived exosomes loaded with KrasG12D siRNA (iExosomes) for treating participants with the KrasG12D mutation and with pancreatic cancer. This is a Phase I study that has not yet started to recruit patients.

The Gustave Roussy and Curie institutes have developed an immunotherapy involving metronomic cyclophosphamide (mCTX), followed by vaccinations with tumor antigen-loaded dendritic cell-derived exosomes (Dex). Phase I trials showed the safety and feasibility of Dex vaccines, but no induction of T cells could be monitored in patients. Since 2007, a new process for the isolation of second generation Dex with improved immune stimulatory capacities was validated; thereby, the group proposed a phase II maintenance immunotherapy study in advanced unresectable NSCLC patients responding to or stabilized after induction chemotherapy with Dex-based treatment, in order to improve the median progression-free survival (PFS) rate at 4 months in these patients.

It is worth emphasizing that all of the products currently under clinical evaluation involve engineered exosomes, and no other types of exosome-like vesicles are currently in the trial.

Nevertheless, although most of the initial clinical trials are ongoing, the use of extracellular vesicles for therapeutic or drug delivery applications may be limited due to the potential for “undesired off-target effects” and also “dilution effects” which may occur following systemic administration, and affect their ability to reach their intended target sites. Recently, in an effort to overcome such off-target effects and exploit the therapeutic potential of extracellular vesicles, they were embedded into implantable biomaterials for local delivery of enzyme prodrug therapy-type (EPT) therapeutics. In other words, extrcellular vesicles were used as smart carriers to stabilize the enzymes in a hydrogel where locally-controlled conversion of a prodrug to the active compound would take place. In this specific case, an anti-inflammatory product was studied, and the vesicles were demonstrated to confer comparable or superior antiinflammatory activity to that of synthetic carriers [177].

### 6.2. Challenges and Future Perspectives

Summarizing the previous parts, the main challenges towards unlocking the potential of exosome-like vesicles, regardless of their type (engineered- exosomes or exosome-mimetics (cellular vesicles or artificial liposomes)), towards the development of novel targeted drug delivery systems with enhanced targeted efficiency, are related to the following factors: (i) the abundance of starting material for their construction and their preparation yield; (ii) the loading efficiency of drugs; (iii) the blood circulation time, assuming that this determines their targeting efficiency; (iv) the inherent targeting efficiency of the selected system, and how this may be affected by various engineering methodologies; (v) methods/roadmaps for scalable, repeatable manufacturing.

For Exosomes and Cellular Vesicles, a first and very important question is whether the use of such vesicles from allogenic sources will initiate immune responses and/or if they will be safe. Providing answers to these questions is particularly urgent for cancer-cell-derived exosomes and cellular vesilces, especially since they are probably the vesicles with the highest organotropism. Despite recent findings about the preferable immune properties of macrophage-derived and other types of exosomes [100,101,102], it is not yet clear which types of exosomes can be safely used as DDSs with broad applicability. A key point in this direction will be to classify the vesicles derived from different sources (different types of cells, biological fluids etc.), in terms of their organotropism, immunogenicity, and biodistribution, following administration by enteric and parenteric routes. Such classifications will be particularly useful for: (a) the election of the optimal type of vesicle source for each specific drug targeting application, and (b) acknowledgement of the challenges that will be encountered in order to achieve the specific goal. In other words, will the vesicles need to be engineered in order to increase their blood circulation time, or their targeting efficiency, or perhaps both?

For Engineered-Exosomes, a challenge remains today to increase production yield. The low yield of isolation of exosomes from cells, together with safety issues, is the main reason for the exploitation of safer and more abundant exosome sources, such as fruit and milk. In the latter case, it is not clear if there are any advantages of such systems compared to targeted-liposomes, especially if i.v. administration will be used. For engineered-exosomes, drug loading also needs to be improved by deeper exploitation of more efficient and perhaps scalable and reproducible microfluidic methods, which have been already explored as methods to increase the yield of exosome-isolation [178]. The development of future lab-on-chip approaches that will include parts for microfluidic isolation of exosomes, and separation by molecular profiling, followed by parts for drug loading by microfluidic mixing, may be the solution for high-yield production of drug-loaded exosomes. However, it remains to be verified if the organotropic exosome-surfaces will be preserved after such manipulations.

Between exosomes and cellular vesicles, the latter seem to have several advantages for clinical applications, the most important of which are: (i) the high yield, and (ii) the simple purification processes required for their production. These two advantages may be the ones that will perhaps facilitate the construction of a roadmap for the rapid manufacturing of engineered cellular vesicles from autologous sources for drug targeting systems. This may be technically possible, providing that the engineering methodologies will be such that the important structural components of the parental cells will be retained on the vesicles produced, for preservation of organotropism, and that, at the same time, any components that may result in detrimental biological effects (e.g., oncogenesis, etc., if the parental cells are tumor cells) will be extracted from the final cellular vesicles produced. In such cases, as suggested before [53], perhaps the high organotropism of such cellular vesicles will prevail, and the required amount of drug for a sustainable therapeutic effect will rapidly reach the target sites, regardless of a potentially low circulation time in the blood. Thereby, no further manipulation of the cellular vesicles will be required to increase their blood circulation time, providing the additional advantage of avoiding any potential side effects caused by hydrophilic coatings which are used in the liposome field (such as PEG).

The development of artificial exosomes or exosome-inspired liposomes may be preferable in order to avoid potential safety problems related with the use of allogenic vesicles from any source, and especially from cancer cells, as well as the low yield and time required for producing vesicles from autologous sources. Additionally, the manufacturing of such systems is already well studied, and microfluidic-based approaches have already been optimized, and ready to use GMP-compatible equipment for scale up production is available. However, although in theory the identification of the protein and lipid components of exosomes may be possible today through the use of advanced proteomic and lipidomic tools, it remains to be verified if such approaches will lead to the development of liposomes with high targeting efficiency, and also if their circulation time will be sufficient for reaching their in vivo targets. Additionally, it is not currently clear how the specific components that are responsible for the high organotropism of exosomes can be identified between their numerous components. Finally, it has never been studied whether cellular vesicles can be used for the same purpose. In fact, perhaps the use of cellular vesicles (and not exosomes) may be preferable, since they are thought to contain the most organotropic-important components of the cell-membrane [133]. Another issue that needs to be explored is whether the proteins are embedded in the membranes with specific conformation, and if this is important for their organotropism.

Finally, several other issues have not yet been considered, such as the stability of engineered-exosomes, engineered-cellular vesicles, and artificial-exosomes, since it is not straightforward to predict how stable the protein parts of their membrane will be during storage. Furthermore, nothing is known about the potential to lyophilize such vesicle dispersions.

## Figures and Tables

**Table 1:** Comparison of the early methods applied for drug loading into engineered extracelluar vesicles (EVs) or exosomes (EXs).

Method	Advantages	Disadvantages	Drug Loaded/Application
Treatment of parental cells with drug	Relatively simple Does not require addition of drug into the system	Low loading efficiency Drugs may be cytotoxic to cells	Paclitaxel (Ptx) [104] Hydrophobic sensitizers (model drug) [141]
Incubation with drug	Simplest method Do not require extra equipement	Low loading efficiency	Curcumin [92,107]; si RNAs [116]; Porphyrins [111]; Catalase [108]; PTX [105]; DOX [129]
Electroporation	Loading with large molecules possible	Disrupts EX integrity siRNA aggregation Low loading efficiency (hydrophobic drugs)	siRNA [112,114]; Porphyrins [111]; DOX [109]; Dextran macromolecules [110]; PTX [105]
Sonication	Increased loading efficiency (compared to other methods) Applicable for small RNAs	Potential deformation of membrane Not efficient for hydrophobic drugs	PTX [105]; Catalase [108]; siRNA, miRNA, ssDNA [114,149]
Extrusion	High drug loading efficiency	Potential deformation of membrane	Porphyrins [111]; Catalase [108]
Freeze/thaw method	Medium loading Fusion of membranes possible [54]	Exosomes may aggregate Low loading Efficiency	Catalase [108]; DOX [129]
Saponin-assisted loading	High drug loading, compared to the other methods used in early reports	Generates pores in EXs Haemolysis/Toxicity concerns [150] Saponin conc. Control & Washing required	Catalase [108]; Porphyrins [111]; DOX [129]

**Table 2:** List of the clinical studies involving the usage of exosomes as DDSs, or related studies. Study Titles are in bold for the most relevant studies.

Status	Study Title	Conditions	Interventions	Phase	NCT Number
**Not yet recruiting**	Allogenic Mesenchymal Stem Cell Derived Exosome in Patients With Acute Ischemic Stroke	Cerebrovascular Disorders	Biological: exosome	Phase 1 Phase 2	03384433
**Enrolling by invitation**	Effect of Plasma Derived Exosomes on Cutaneous Wound Healing	Ulcer	Other: plasma-derived exosomes	Early Phase 1	02565264
**Active, not recruiting**	**Study Investigating the Ability of Plant Exosomes to Deliver Curcumin to Normal and Colon Cancer Tissue**	Colon Cancer	curcumin Curcumin conjugated with plant exosomes	Phase 1	01294072
**Recruiting**	Dendritic Cells-Derived Exosomes in Human Sepsis	Sepsis	Drug: Antibiotics		02957279
**Not yet recruiting**	Plant Exosomes and Patients Diagnosed With Polycystic Ovary Syndrome (PCOS) 17	Polycystic Ovary Syndrome	Ginger exosomes Aloe exosomes Placebo	Not Applicable	03493984
**Unknown**	Effect of Microvesicles and Exosomes Therapy on β-cell Mass in Type I Diabetes Mellitus (T1DM)	Diabetes Mellitus Type 1	Biological: MSC exosomes.	Phase 2 Phase 3	02138331
**Not yet recruiting**	**iExosomes in Treating Participants With Metastatic Pancreas Cancer With KrasG12D Mutation**	KRAS NP_004976.2:p.G12D Metastatic Pancreatic Adenocarcinoma	Drug: Mesenchymal Stromal Cells-derived Exosomes with KRAS G12D siRNA	Phase 1	03608631
**Recruiting**	MSC-Exos Promote Healing of MHs	Macular Holes	Biological: exosomes derived from mesenchymal stem cells (MSC-Exo)	Early Phase 1	03437759
**Active, not recruiting**	Edible Plant Exosome Ability to Prevent Oral Mucositis Associated With Chemoradiation Treatment of Head and Neck Cancer	Head and Neck Cancer Oral Mucositis	Dietary Supplement: Grape extract Drug: Lortab, Fentanyl patch, mouthwash	Phase 1	01668849
**Completed**	**Trial of a Vaccination With Tumor Antigen-loaded Dendritic Cell-derived Exosomes**	Non Small Cell Lung Cancer	Biological: Dex2	Phase 2	01159288
